# Gα_q_ modulates the energy metabolism of osteoclasts

**DOI:** 10.3389/fcimb.2022.1016299

**Published:** 2023-01-09

**Authors:** Sushmita Chakraborty, Bianca Handrick, Dayoung Yu, Konrad A. Bode, Anna Hafner, Judith Schenz, Dominik Schaack, Florian Uhle, Taro Tachibana, Shigeki Kamitani, Thomas Vogl, Katharina F. Kubatzky

**Affiliations:** ^1^ Department of Infectious Diseases, Medical Microbiology and Hygiene, Heidelberg University, Heidelberg, Germany; ^2^ Department of Transplant Immunology and Immunogenetics, All India Institute of Medical Sciences, New Delhi, India; ^3^ Department of Anesthesiology, Heidelberg University Hospital, Heidelberg, Germany; ^4^ Department of Chemistry and Bioengineering, Graduate School of Engineering, Osaka Metropolitan University, Osaka, Japan; ^5^ Department of Nutrition, Graduate School of Human Life and Ecology, Osaka Metropolitan University, Osaka, Japan; ^6^ Institute of Immunology, University Hospital Münster, Münster, Germany

**Keywords:** pasteurella mulfocida toxin, mitochondria, immunometabolism, OPA1, STAT3, Gαq, osteoclast, rheumatoid arthritis

## Abstract

**Introduction:**

The bacterial protein toxin *Pasteurella multocida* toxin (PMT) mediates RANKL-independent osteoclast differentiation. Although these osteoclasts are smaller, their resorptive activity is high which helps in efficient destruction of nasal turbinate bones of pigs.

**Methods:**

The proteome of bone marrow-derived macrophages differentiated into osteoclasts with either RANKL or PMT was analysed. The results were verified by characterizing the metabolic activity using Seahorse analysis, a protein translation assay, immunoblots, real-time PCR as well as flow cytometry-based monitoring of mitochondrial activity and ROS production. A Gαq overexpression system using ER-Hoxb8 cells was used to identify Gαq-mediated metabolic effects on osteoclast differentiation and function.

**Results:**

PMT induces the upregulation of metabolic pathways, which included strong glycolytic activity, increased expression of GLUT1 and upregulation of the mTOR pathway. As OxPhos components were expressed more efficiently, cells also displayed increased mitochondrial respiration. The heterotrimeric G protein Gαq plays a central role in this hypermetabolic cell activation as it triggers mitochondrial relocalisation of pSerSTAT3 and an increase in OPA1 expression. This seems to be caused by a direct interaction between STAT3 and OPA1 resulting in enhanced mitochondrial respiration. Overexpression of Gαq mimicked the hypermetabolic phenotype observed for PMT-induced osteoclasts and resulted in higher glycolytic and mitochondrial activity as well as increased bone resorptive activity. In addition, rheumatoid arthritis (RA) patients showed an increase in *GNAQ* expression, especially in the synovial fluid.

**Discussion:**

Our study suggests that Gαq plays a key role in PMT-induced osteoclastogenesis. Enhanced expression of *GNAQ* at the site of inflammation in RA patients indicates its pathophysiological relevance in the context of inflammatory bone disorders.

## 1 Introduction

Bone remodelling is a continuous process throughout life and various soluble factors and cells influence this process. Changes in the microenvironment of bone during an infection or inflammatory processes lead to bone loss due to excessive formation and function of osteoclasts. Osteoclasts are bone resorbing haematopoietic cells that can be differentiated from their respective monocyte precursor cells through the two cytokines macrophage colony stimulating factor (M-CSF) and receptor activator of NF-kB ligand (RANKL). Pro-inflammatory cytokines such as IL-6, TNF-α and IL-1β can act as additional stimuli that enhance osteoclast formation (Shaw and Gravallese, 2016). Therefore, auto-inflammatory pathologies such as rheumatoid arthritis (RA) or chronic inflammation, e.g. osteomyelitis, are often accompanied by a decrease in bone density. During differentiation, preosteoclasts fuse and form polycaryons before becoming mature osteoclasts. Osteoclasts are defined as multi-nucleated cells that stain tartrate resistant acidic phosphatase (TRAP)-positive and resorb bone. To enable cellular fusion and bone resorption, osteoclasts increase their mitochondria to meet the energy demand (Arnett and Orriss, 2017). However, our understanding of the changes in metabolic activity during osteoclast differentiation is still limited. In addition to TCA cycle activation and OxPhos activity, glycolysis is involved and glucose transporter GLUT1 is upregulated in a RANKL-dependent manner (Indo et al., 2013). The importance of mitochondrial activity is highlighted by the finding that inhibition of mitochondria through deletion of the complex I component Ndufs4 prevents osteoclast differentiation and shifts macrophages towards M1 activation and inflammation (Jin et al., 2014). Due to the close relationship between macrophages and osteoclasts it can be expected that changes in macrophage activity, for example during bacterial bone infections or auto-inflammatory diseases, will impact the ability of macrophages to differentiate into osteoclasts and shape the activity of the resulting osteoclast, respectively. The possibility to manipulate cell metabolism is by now well-established in cancer therapy, but has only recently been discovered as an interesting option in diseases like RA, systemic lupus erythematosus or osteoarthritis (Onuora, 2016; Sanchez-Lopez et al., 2019; Takeshima et al., 2019; Mao et al., 2020).

The central importance of heterotrimeric G proteins for bone formation became clear through the link between fibrous dysplasia of bone, where postnatal skeletal stem cells generate skeletal lesions at one or even multiple sites of the skeleton. This is caused by the constitutive activation of the alpha subunit of the heterotrimeric protein Gs through a point mutation (Riminucci et al., 1999). More recently, the role of other G proteins was investigated and specific functions could be attributed to some. Gα13 for example is a negative regulator of osteoclast fusion that restricts osteoclast size (Wu et al., 2017; Nakano et al., 2019), whereas Gα12 enhances osteoclast formation and activity by positively regulating NFATc1 signalling and bone resorption (Song et al., 2018). The role of Gαq in osteoclastogenesis has not been studied so far. However, GPR91, the receptor for succinate, which signals *via* Gαq, was recently shown to be important in pathological bone loss in RA (Littlewood-Evans et al., 2016). As the bacterial toxin *Pasteurella multocida* toxin (PMT) is a strong activator of Gαq, we used PMT as a tool to investigate the role of mitochondrial Gαq during osteoclast differentiation.

PMT is known to be the causative agent of *Pasteurella multocida* that causes atrophic rhinitis in pigs and the severe destruction of nasal bone, is produced by toxigenic *Pasteurella multocida* strains (Kubatzky, 2012). Deamidation and subsequent activation of heterotrimeric G proteins, such as Gαq/11, Gα12/13 and Gαi, causes cytoskeletal rearrangement, proliferation, protection from apoptosis and the differentiation of macrophages into osteoclasts (Kubatzky et al., 2013). PMT causes osteoclastogenesis RANKL-independently through Gαq-dependent activation of nuclear factor of activated T cells c1 (NFATc1) and NF-kB-mediated production of pro-inflammatory cytokines (Strack et al., 2014; Chakraborty et al., 2017). Our proteome analysis of classical and toxin-derived osteoclasts showed an overrepresentation of genes involved in glycolysis and metabolic pathways in PMT-stimulated samples which was verified by Seahorse analyses of glycolysis and extracellular acidification (ECAR) measurements. Overexpression of Gαq is central for serine phosphorylation and mitochondrial localisation of the signal transducer and activator of transcription (STAT3) and increased expression of the large dynamin-like GTPase OPA1 resulting in more efficient mitochondrial cristae formation, enhanced ETC expression and increased resorptive activity.

In summary, we show that Gαq plays an important role in the induction of a hypermetabolic phenotype in macrophages characterized by an upregulation of glycolytic activity as well as an enhanced capacity for oxygen consumption facilitating osteoclast differentiation and activity. Enhanced expression of Gαq was also observed in cells from synovial fluid of RA patients indicating its influence in disease pathogenesis.

## 2 Materials and methods

### 2.1 Reagents

Cell culture reagents were purchased from Anprotec (Bruckberg, Germany) for media, Biochrom GmbH (Berlin, Germany) for FCS, PAN Biotech for β-mercaptoethanol and Sigma-Aldrich for oestradiol (St. Louis, USA). PCR primers were purchased from Biomers (Ulm, Germany). The cytokines M-CSF and RANKL were bought from BioTechne (Abington, UK) and IL-6 from Miltenyi (Bergisch-Gladbach, Germany). PMT was produced recombinantly as described previously (Chakraborty et al., 2017). ER-Hoxb8 cells were kindly given by Hans Häcker (University of Utah). A detailed list of the reagents including antibodies and primers can be found in the **supplementary key resource table**.

### 2.2 Mice and BMDM culture

6- to 8-week-old female C57BL/6J (Janvier Labs, LeGenest St. Isle, France) mice were bred under SPF conditions and sacrificed in accordance with the animal care guideline approved by German animal welfare authorities. Bone marrow cells were isolated from mice femur and tibia, and washed with 1x PBS. Cells were differentiated into bone marrow-derived macrophages (BMDMs) in high glucose Dulbecco’s medium supplemented with 10% FCS, 1% penicillin/streptomycin, and 50 µM ß-mercaptoethanol (DMEM) and M-CSF derived from L929 cells. BMDM were harvested on day 6 and were stimulated with 25 ng/ml M-CSF, 50 ng/ml RANKL, 50 ng/ml IL-6 or 1 nM PMT as indicated.

### 2.3 Retroviral transduction of ER-Hoxb8 cells

pMX-Gαq-IRES-CD4 or pMX-IRES-CD4 (mock) was transiently transfected into Phoenix-Eco cells using ProFection^®^ Mammalian Transfection System (Promega, Walldorf, Germany) according to the manufacturer’s instructions. Retroviral supernatant was collected 2 days post transfection and used for infection of ER-Hoxb8 cells (Wang et al., 2006). 5x10^5^ ER-Hoxb8 cells were centrifuged with 750 µl of virus supernatant and 16 ug polybrene at 1100 rpm (37°C) for 2 hours. Cells were further cultured in RPMI 1640 medium with 10% FCS, 1% penicillin/streptomycin, 5% GM-CSF supernatants (Zal et al., 1994), 1 µM β-oestradiol. Additional 2.5 g/L glucose was supplemented in RPMI 1640 medium for efficient cell viability. After 48 h, infected ER-Hoxb8 cells were selected with 8 µg/ml puromycin. Transduction efficiency was confirmed by huCD4 expression based on flowcytometric analysis.

### 2.4 Osteoclast differentiation and TRAP staining

For differentiation experiments, GM-CSF and oestradiol were removed by washing and 7x10^4^ ER-Hoxb8 cells were resuspended in DMEM medium before seeding in 48-well plates. Cells were stimulated with 25 ng/ml M-CSF, 50 ng/ml RANKL; every 3 days half of the culture medium was replenished with medium containing M-CSF and RANKL. Multi-nucleated cells were observed under a light microscope Axiovert 25 typically after 4 to 5 days in culture. Cells were fixed and stained using the Leukocyte Acid Phosphatase (TRAP) kit according to the manufacture’s recommendations. TRAP-positive cells with more than 3 nuclei were counted as mature osteoclasts. For confocal laser scanning microscopy, cells were plated on Poly-D-Lysin-coated coverslips in a 24-well plate at a density of 2 x 10^5^ cells/well. Active TRAP was detected using fluorescence-labelled ELF 97 phosphatase substrate (Thermo Fisher, Waltham, USA) as described previously (Filgueira, 2004; Kloos et al., 2015). The actin cytoskeleton was visualized suing TRITC-conjugated Phalloidin (Sigma Aldrich, St. Louis, USA) and nuclei with DAPI (Thermo Fisher, Waltham, USA). Cells were fixed with 3.7% paraformaldehyde washed with PBS and permeabilised with 0.2% TritonX100 for 20 min, washed three times with PBS and incubated with the various stains. To visualize the cells, a confocal laser scanning microscope (Leica) was used with a 1.4 NA 63× immersion oil objective. Pictures were taken at 400 Hz with 4× line and frame average.

### 2.5 Bone resorption assay

2 x 10^5^ ER-Hoxb8 or ER-Hoxb8-Gαq cells were seeded per well in a 24-well plate and stimulated with 25 ng/ml M-CSF, 50 ng/ml RANKL for 3 days. For the bone resorption assay, cortical bovine bone slices were used (Boneslices.com, Jelling, Denmark). Bone slices were washed with medium several times in order to remove the alcohol and placed in the 96-well plate. After 3 days of stimulation, cells were gently detached and transferred onto bone slice in a 96-well plate. Cells were kept on bone for 15 days; every third day, half of the medium was replenished. After removing the cells from the bone slices, pits were stained with freshly prepared 0.1% toluidine blue. Pit formation on bone slices was analysed with a Rebel Microscope (ECHO, San Diego, USA).

### 2.6 NTX ELISA

Cell supernatants collected on day 9 during the bone resorption assay were used to determine the levels of secreted N-telopeptide of type 1 collagen. Experimental details were followed using the manufacturer’s protocol (Gentaur, Kampenhout, Belgium). Cell supernatants were added along with NTX biotinylated antibody and Streptavidin-HRP into a pre-coated well with NTX antibody for 1 h at 37°C. After 1 hour, wells were washed 5 times with wash buffer. The substrate solution was added to develop the colour and the reaction was stopped using stop solution. The absorbance was measured at 450 nm using a CLARIOstar instrument (BMG Labtech, Offenburg, Germany).

### 2.7 Real-time PCR

Total RNA was isolated from cells using innuPREP RNA Mini Kit 2.0 (Analytik Jena, Jena, Germany). RNA isolation was performed in accordance with the provided manual and quantified by a Nanodrop.

500 ng of total RNA was converted to cDNA using a cDNA synthesis kit. Real-time PCR analysis was further performed on a StepOnePlus Real-Time PCR System from Applied Biosystems using 2x qPCRBIO SyGreen Mix Hi-ROX (PCR Biosystems, London, UK). Specific primer pairs can be found in the key resources table. Relative gene expression of target genes was calculated in comparison to the Ct value of the house-keeping gene *Rsp29* using 2^-[Ct(target gene)−Ct(reference gene)]^.

### 2.8 Expression of *GNAQ* and *OPA1* in human samples

The study was conducted with approval from the Institute Ethics Committee (IEC-490/01.09.2017). After obtaining informed consent, patients with RA were recruited from Orthopaedics OPD of AIIMS, New Delhi [Age in years (Mean ± SD): 41 ± 11 years; Sex: Female]. Diagnosis was made on the basis of ACR criteria 1987. For this study, we collected synovial fluid (SF) and autologous peripheral blood (PB) from RA patients with active disease. We also collected peripheral blood of RA patients with low disease condition (Under treatment) [Age in years (Mean ± SD): 45 ± 6 years; Sex: Female]. Healthy controls (HC) collected for the studies, were free from any acute or chronic ailment and were not on medication at the time of enrolment. Peripheral blood from HC was collected [Age in years (Mean ± SD): 35 ± 5 years; Sex: Female]. Specimens were collected in heparinized tubes. Synovial Fluid mononuclear cells (SFMC) and Peripheral blood mononuclear cells (PBMCs) were isolated using Lymphoprep (Axis-Shield, Dundee, UK), density gradient centrifugation. Cells were resuspended in RPMI-1640 containing L-Glutamine, and HEPES supplemented with, 10% FBS, 100 U/ml penicillin and 100 µg/ml streptomycin. A total of 5×10^6^ cells were seeded per well of 6-well plate. Cells were kept in humidified 5% CO_2_ incubator at 37°C for 24 h. After 24 h, non-adherent cells were removed by washing the wells with PBS three times. Adherent cells were processed for expression studies. RNA was extracted using GeneJET RNA Purification Kit (Thermo Scientific, Waltham, USA), according to the manufacturers’ protocol. cDNA was prepared by using Revert Aid First strand cDNA synthesis kit (Thermo Scientific, Waltham, USA). Quantitative RT-PCR was performed using Powerup Sybr Master Mix. RT-PCR was performed using the QuantStudio 5 Real-Time PCR Systems (Applied Biosystems, Waltham, USA). An initial enzyme activation step of 2 min at 50°C and denaturation step of 5 min at 95°C was followed by amplification for 40 cycles at 95°C for 15 s and at 56°C for 1 min. As normalization control *ACTB* was used. Relative gene expression of target genes was calculated in comparison to Ct value of normalizing control using 2^-[Ct(target gene)−Ct(reference gene)]^.

### 2.9 Immunoblotting analysis

Cells were lysed using RIPA buffer (1% NP-40, 0.25% deoxycholate, 50 mM Tris pH7.4, 150 mM NaCl, 1 mM EDTA, 1 mM Na_3_VO_4_) supplemented with a Phosphatase and Protease-Inhibitor Cocktail (Roche, Mannheim, Germany). Cell lysates were obtained after shaking for 45 min at 4°C, followed by centrifugation for 20 min at 15,000 rpm at 4°C. About 10 µg of proteins were loaded on a Tris Glycine 4-20% polyacrylamide gel (Anamed, Groß-Bieberau, Germany), and then transferred to the nitrocellulose blotting membrane *via* semi-dry blot system. Membranes were blocked in 1x blocking buffer for 30 min at r.t. and incubated with the desired primary antibody overnight at 4°C. Probed bands were detected by the appropriate HRP-conjugated secondary antibody upon incubation for 1 hour at r.t. Membranes were developed using the ECL substrate WESTAR ηC Ultra 2.0 (Cyanagen, Bologna, Italy) and visualized on ChemoStar image (Intas Science Imaging, Göttingen, Germany).

For Co-IP studies, 1.5x10^7^ stimulated BMDMs were lysed in a 1x Brij buffer with 1% Brij97, 150 mM NaCl, 20 mM Tris pH7.4, 1 mM EDTA, 1 mM MgCl_2_, 1mM Na_3_VO_4_, and 10% Glycerol with a Phosphatase and Protease-Inhibitor Cocktail. Collected samples were incubated with protein A/G plus agarose beads and anti-mouse OPA1 antibody overnight at 4°C. Beads were gently washed twice with Brij buffer and once with TNE buffer for 15 s before SDS-PAGE. ImageJ was used to assess protein expression levels.

### 2.10 Mitochondrial fractionation

Mitochondrial fractionation was performed based on sequential centrifugation using a Mitochondrial Isolation Kit for Mammalian Cells according to manufacturer’s instructions (Thermo Fisher, Waltham, USA). 2x10^7^ of BMDMs were lysed with isolation reagents supplemented with 20x Protease inhibitor. Samples were homogenized using a Dounce tissue grinder. All centrifugation steps were conducted at 4°C. Homogenates were centrifuged for 10 min at 3000 rpm and supernatants were transferred for additional centrifugation for 15 min at 12,000 rpm to obtain cytosolic proteins from the supernatant. The pellet was washed for 5 min at 12,000 rpm and resuspended in 2% CHAPS buffer with 25 mM Tris-HCl, 0.15 M NaCl, pH7.2. Mitochondrial fractions as supernatants were isolated after centrifugation for 2 min at 13,000 rpm.

### 2.11 Mitochondrial DNA copy number

Total DNA was extracted using DNAeasy Blood and Tissue Kit (Qiagen, Hilden, Germany) following the provided protocol. 10 ng of DNA was amplified by RT-PCR as described above. Relative mitochondrial DNA contents were normalized to the level of nuclear DNA (see primer sequences in the key resources table). Relative mitochondrial DNA copy was calculated as 2x2^(nucleus Ct-mitochondria Ct)^.

### 2.12 Translation assay

Translational activity was achieved by the methionine analogue of L-homopropargylglycine (HPG) incorporation *via* Click-iT^®^ HPG Alexa Fluor^®^ 488 Protein Synthesis Assay Kit (Thermo Scientific, Waltham, USA). 1x10^6^ of BMDMs in a 24-well plate were stimulated for 6 hours, and 50 µM HPG was added 30 minutes before the end of stimulation. Samples were fixed in 4% PFA and permeabilized with 0.5% Saponin. Click-iT^®^ reaction cocktail including Alexa Fluor 488 dye was added in each well for 40 minutes at room temperature in the dark. Protein synthesis was evaluated by measuring FITC channel for Alexa Fluor 488 on BD FACSDiva software using a BD FACSCanto Cytometer (BD Biosciences, Heidelberg, Germany). The data show MFI values normalised to the M-CSF-treated control cells.

### 2.13 Seahorse assay

Live cell metabolic analyses were performed measuring extracellular acidification rate (ECAR) and oxygen consumption rate (OCR) using Seahorse XFp Analyzer (Agilent Technologies, Santa Clara, USA). 4x10^5^ BMDM per well were seeded in 96-well format and incubated for 3 days. One hour before the measurements, the growth medium was replaced by Seahorse XF Base Medium (without Phenol Red) supplemented with 5 mM HEPES, 1 mM sodium pyruvate, 2 mM L-glutamine, only for assays analysing mitochondrial respiration, 10 mM D(+)Glucose. Thereafter, cells were incubated at 37°C without CO_2_ until start of the assay. After three baseline measurements (each measuring point comprises 3 min mixing and 3 min measuring) 2 µM oligomycin, 1.5 µM Carbonyl cyanide–4 (trifluoromethoxy) phenylhydrazone (FCCP), and 0.5 µM rotenone/antimycin A (all three from Seahorse XFp Cell Mito Stress Test Kit) were added sequentially to characterize mitochondrial respiration. To determine glucose uptake and glycolytic function 10 mM glucose, 2 µM oligomycin, and 50 mM 2–Deoxy-D–glucose (2-DG) (all three from Seahorse XFp Glycolysis Stress Test Kit) were added sequentially. OCR and PER were calculated using the Wave 2.6.0 software and parameters were calculated according to the manufacturer’s instruction using Seahorse XF Cell Mito Stress Test Report Generator 3.0.11 and Seahorse XF Glycolysis Stress Test Report Generator 4.0.

### 2.14 Mitochondrial activity

For FACS analysis, 1×10^6^ BMDM or ER-Hoxb8 cells were used per sample. Mitochondrial activity was determined by staining of cells for 30 minutes with 100 nM MitoTracker Deep Red (Cell Signaling Technologies, Leiden, The Netherlands). Cells were then washed three times with 1x PBS and the mean fluorescence intensity (MFI) was measured in the APC channel.

### 2.15 2-DIGE

A difference-in-gel electrophoresis (DIGE) technique was used to investigate proteome differences between PMT-stimulated and M-CSF/RANKL and M-CSF-stimulated cells (n=3 biological replicates with 3 technical replicates). 3×10^7^ cells were seeded to generate osteoclasts from BMDM. Cells were washed before lysis in 150 µl THC buffer. 100 μg of protein samples were stained using the Amersham CyDye DIGE Fluor minimal dye labelling kit according to the manufacturer’s instructions. All samples were stained once with Cy5 and once with Cy3, while the normalization control (all samples combined) was stained with Cy2. The samples to be compared (Cy2 and Cy3 stained) were then mixed with the Cy2-stained normalization control and separated two-dimensionally according to isoelectric point and molecular weight. The samples were applied to IPG strips and separated in the first dimension with the IPGphor 3 system (GE Healthcare, Chicago, USA) according to their isoelectric focusing (20 C, I < 25 μA). After equilibration of the IPG strips in reduction buffer and alkylation buffer, proteins were separated according to their molecular weight in the second dimension using a gradient gel (8%-15%) in an Ettan DALT II system (GE Healthcare, Chicago, USA). The gels were then scanned at the different wavelengths using a Typhoon 9400 Laser Scanner (GE Healthcare, Chicago, USA) and analysed using the DeCyder 2D software. After quantification of data derived from 6 gels (3 biological replicates, with three technical replicates), the most highly regulated spots were automatically picked with an Ettan Spot Picker (GE Healthcare, Chicago, USA) and transferred to a 96-well plate. The spots were analysed by mass spectrometry (LTQ Orbitrap XL mass, Thermo Fisher Scientific, Waltham, USA) by Dr. Martina Schnölzer (DKFZ, Heidelberg).

### 2.16 Total ROS assay

5×10^4^ cells were seeded in black 96-well plates and stimulated as indicated. On day 3, the cells were processed according to the manufacturer’s protocol (Thermo Scientific, Waltham, USA). Fluorescence at 520 nm was measured using a FluoStar Optima (BMG Labtech, Offenburg, Germany).

### 2.17 GSH-Glo Assay

For this assay (Promega, Walldorf, Germany), 2×10^4^ cells were seeded in a 96-well plate and stimulated as indicated. On day 3, the cells were processed according to the manufacturer’s protocol. Luminescence was measured in white 96-well plate using a LumiStar Optima (BMG Labtech, Offenburg, Germany). The glutathione (GSH) concentration was calculated using a standard curve (16 μM to 0.125 μM).

### 2.18 Data analysis

All data are presented as mean ± SD. Statistical analyses were carried out using GraphPad Prism 9.0 software as indicated in the respective figure legend. The statistical significance is displayed as **** for p < 0.0001, *** for p from 0.0001 to 0.001, ** for p from 0.001 to 0.01, * for p 0.01 to 0.05 and not significant for p ≥ 0.05.

## 3 Results

### 3.1 Distinct proteome between PMT and M-CSF/RANKL-induced osteoclasts

**Figure 1:**
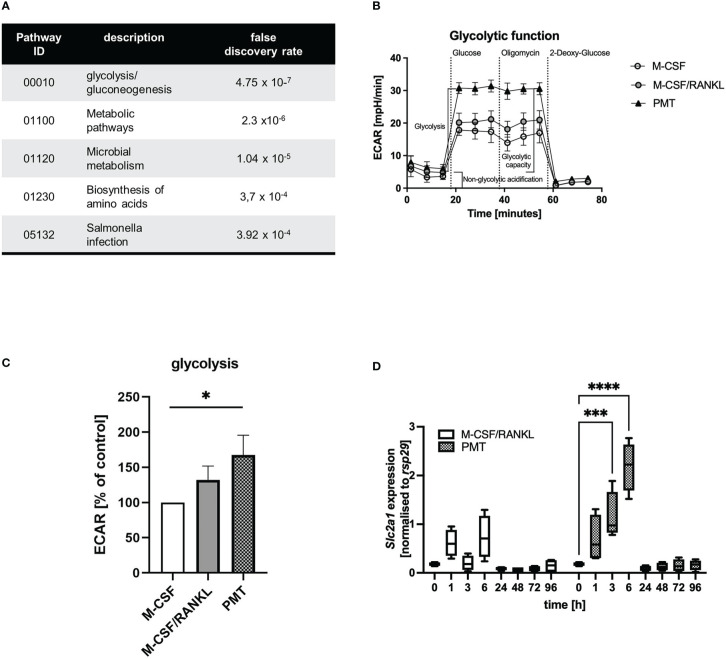
Comparison of osteoclast glycolytic activities during differentiation. **(A)** Proteins that were upregulated with PMT (10 most highly picked, results obtained for 7, see **Supplementary Table**) were subjected to a Panther GO analysis. **(B, C)** Glycolysis of BMDM differentiated for 3 days with M-CSF, M-CSF/RANKL or PMT was investigated in a Seahorse glycolysis assay with quantification of ECAR (n=3). ECAR results were normalised to the M-CSF-treated control. For statistical analysis a Friedman test was used on raw data. **(D)** BMDM were treated with M-CSF/RANKL or PMT and samples were taken at the indicated time-points to quantify the expression of *Slc2a1* by RT-PCR (n=4). Statistical analysis was performed by 2-way ANOVA.

We recently investigated the mechanism of PMT-induced osteoclastogenesis from bone marrow-derived macrophages (Chakraborty et al., 2017). Here, we showed that 1 nM of PMT induced formation of approximately 600-800 osteoclasts, whereas M-CSF/RANKL (25 and 50 ng/ml, respectively) induced approximately 700-1100 osteoclasts. Despite the significant reduction of osteoclast numbers between M-CSF/RANKL and PMT, a striking difference in cellular morphology and a decreased size of the toxin-derived osteoclasts, we found the functional ability of PMT-derived and classical osteoclasts to resorb bone to be comparable (Chakraborty et al., 2017) (**Supplementary Figures 1A, B**). Generally, reduced fusion is linked to ineffective resorption (Mizoguchi et al., 2013), but the drastic effects of PMT in pigs suffering from atrophic rhinitis argue against this (Wilkie et al., 2012). We therefore tried to understand how PMT osteoclasts compensate for the loss of fusogenic activity and performed an analysis of the osteoclast proteome using osteoclasts derived from M-CSF/RANKL and PMT-treated BMDM (**Supplementary Figure 1C**). For an initial analysis the spots that were most highly upregulated by PMT were identified and analysed by mass spectrometry (**Supplementary Figure 1D**). Performing a GO enrichment analysis we could show that metabolic pathways, the biosynthesis of amino acids as well as glycolysis and gluconeogenesis events (Panther analysis of KEGG pathways) were significantly overrepresented after PMT treatment (**Figure 1A**; **Supplementary Figure 1E, F**; **Supplementary Table**).

### 3.2 Differential regulation of glycolytic pathways

To verify the computational results, we analysed the glycolytic pathway and performed a Seahorse glycolytic stress test assay on day 3 of differentiation (**Figure 1B**). Indeed, in PMT-treated cells, the extracellular acidification rate (ECAR) increased significantly after glucose addition compared to M-CSF treated cells, corresponding to higher glycolysis (**Figure 1C**). M-CSF/RANKL-treated osteoclasts also showed enhanced glycolytic activity, although less prominent than PMT (**Figures 1B, C**). Total glycolytic capacity and glycolytic reserve were not altered significantly (**Supplementary Figures 2A, B**) and due to the maximal usage of glycolytic enzymes after the addition of glucose, no further increase in ECAR was observed by addition of oligomycin in all cell types. This was corroborated by our finding that glucose transporter *Slc2a1* transcription was induced at early time points by PMT but only weakly by M-CSF/RANKL (**Figure 1D**). Because an increase in glycolysis is associated with anabolic pathways and a subsequent decrease in intracellular ATP levels, we investigated the expression of glyceraldehyde-3-phosphate dehydrogenase (GAPDH) and activation of AMPK. As suggested by the initial proteomic data, GAPDH was expressed stronger in PMT-treated cells (**Supplementary Figure 2C**). RANKL can induce AMPK activation through phosphorylation on Thr172 of its alpha-subunit (Lee et al., 2010). However, while pAMPK decreased during the course of classical osteoclastogenesis, pAMPK levels were higher im PMT-treated cells on days 3 and 7.

In RAW 264.7 macrophages, the mTOR pathway is essential for PMT-induced osteoclast formation, but dispensable for cytokine production and cellular viability (Kloos et al., 2015). We investigated the ability of PMT to activate the mTOR pathway, as mTOR is a central switch that regulates cellular metabolic activity. Compared to the untreated control, mTOR shifted to a higher molecular weight in M-CSF/RANKL and PMT-treated samples suggesting additional post-translational modifications (**Supplementary Figure 2D**). The levels of pmTOR were high in M-CSF/RANKL treated cells on day 1, but were lower compared to PMT-treated cells on days 3 and 7. Both downstream effector molecules, 4EB-P1 and p70S6K were phosphorylated stronger in PMT-treated BMDM on days 3 and 7 compared to M-CSF/RANKL-treated cells on the same day (**Supplementary Figure 2D**).

### 3.3 Mitochondrial activity during osteoclastogenesis

**Figure 2:**
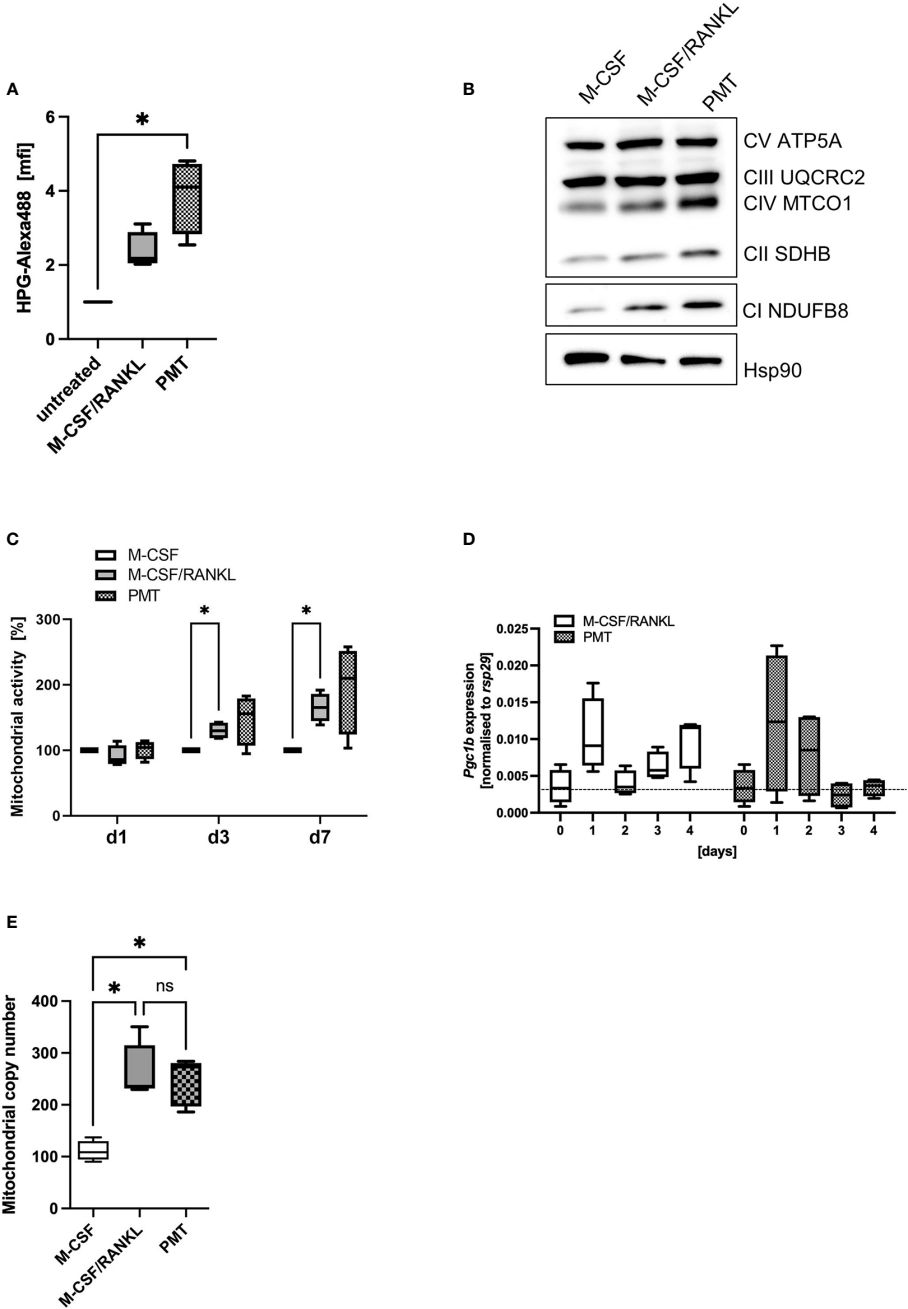
Mitochondrial activity during osteoclastogenesis. **(A)** Analysis of cellular translational activity using an HPG assay, were the methionine analogue HPG is coupled to Alexa488 on newly synthesised proteins (n=4). **(B)** Expression of OxPhos proteins on day 3 of differentiation of BMDM. Hsp90 was used as control (n=3). **(C)** To quantify mitochondrial activity, differentiating BMDM were incubated at the indicated days with Mitotracker Deep Red and fluorescence intensity was quantified and normalised to the M-CSF control (n=4). **(D)** Expression of *Pgc1b* was investigated by RT-PCR in differentiating BMDM and normalised to the house-keeping gene *rsp29* (n=4). **(E)** BMDM were differentiated into OCs for 4 days before preparing genomic DNA. Mitochondrial copy number was determined by RT-PCR comparing the expression of a mitochondria specific sequence with nuclear DNA (n=5). Statistical analysis was done using a Friedman test for **(A)** on raw data before normalisation, 2-way ANOVA for **(C)** and **(D)** and Kruskal-Wallis for unmatched analysis for **(E)**.

A central function of mTOR activation is the activation of anabolic pathways that sustain translational activity. To investigate if the higher phosphorylation levels and subsequent inactivation of p4E-BP1 by PMT would lead to initiation of elF4-mediated protein translation, we performed a metabolic pulse chase experiment with the methionine analogue HPG coupled to Alexa488. **Figure 2A** shows that both, M-CSF/RANKL and PMT increased protein translation (day 3), however, only in the case of PMT treatment the effect reached statistical significance. Morita et al. had reported that the mTOR-activated translation initiation factor elF4 is a central mediator of the translation of ETC components (Morita et al., 2015). This prevents that mTOR-mediated glycolysis and anabolic activity will eventually deprive the cell of ATP and allows the cell to switch back again to OxPhos when needed. We therefore investigated the expression of OxPhos proteins and found an increase in protein expression correlating to the increase in translational activity (**Figure 2B**). Inhibition of mTORC1 activity by rapamycin decreased the PMT-mediated increase in OxPhos proteins (**Supplementary Figure 3**). In addition, mTOR also regulates mitochondrial function which instigated us to measure the activity of mitochondria using the Mitotracker Deep Red FM dye and we found that PMT-treated cells showed a stronger mitochondrial activity at all time points (**Figure 2C**).

Mitochondrial biogenesis is an important part of RANKL-mediated osteoclast differentiation. Therefore, we investigated whether the increased OxPhos expression of PMT osteoclasts might go along with an enhanced formation of mitochondria. Peroxisome proliferator-activated receptor gamma coactivator 1-beta (*Pgc1b*), the gene encoding a transcription factor responsible for mitochondrial biogenesis, was upregulated in both types of differentiating osteoclasts, although statistical significance was not reached (**Figure 2D**). However, we observed a significant increase in the mitochondrial copy number in PMT-treated and M-CSF/RANKL treated cells with no significant difference between PMT- and M-CSF/RANKL osteoclasts (**Figure 2E**).

### 3.4 STAT3 is an interaction partner of OPA1 in mitochondria

**Figure 3:**
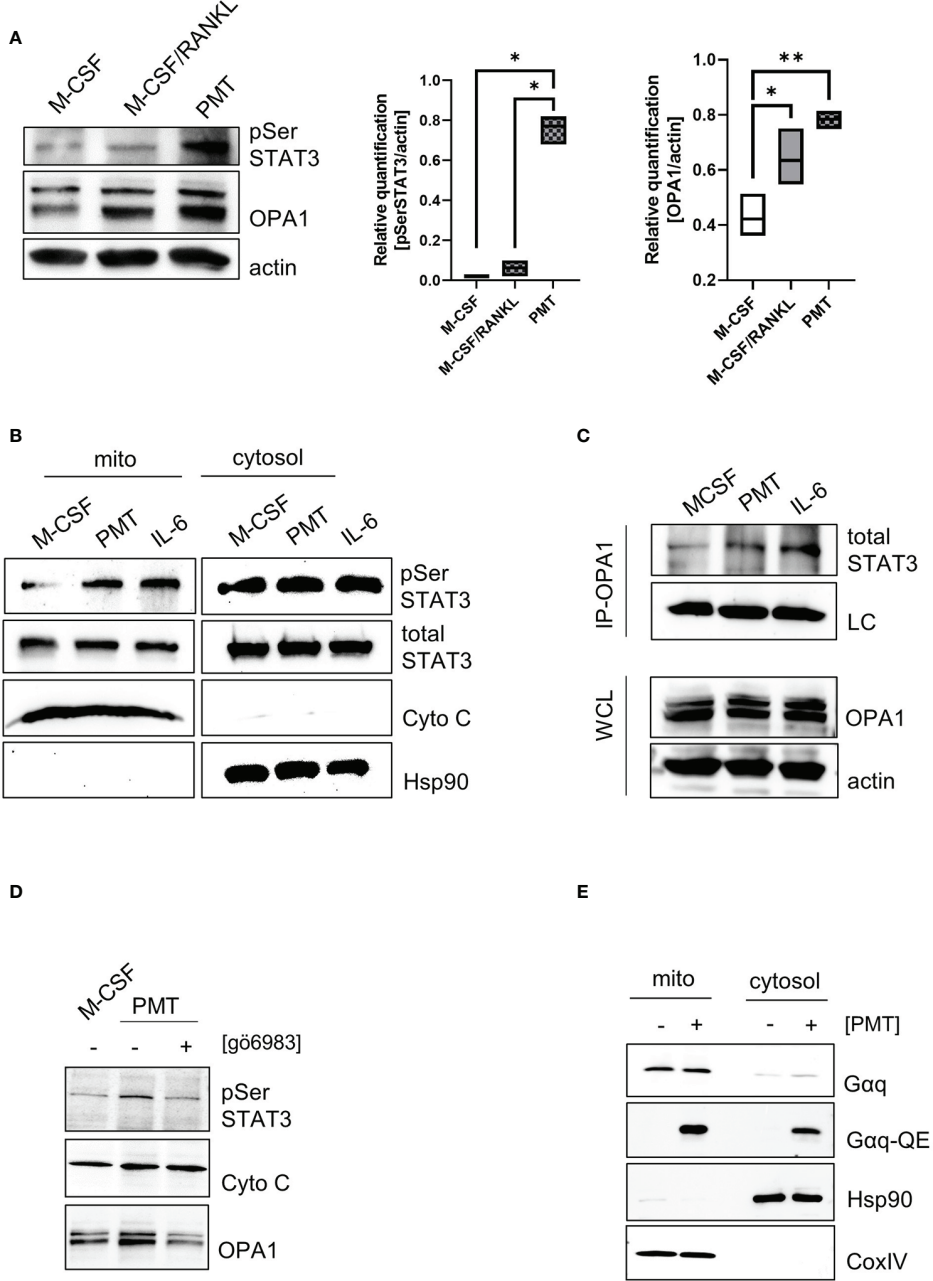
Gαq supports STAT3 mitochondrial translocalisation and stabilises OPA1 expression. **(A)** Whole cell lysates showing STAT3 serine phosphorylation and OPA1 expression on day 3 of differentiation. Quantification of pSer STAT3 and Opa1 levels relative to actin which was used as a loading control (n=4). Statistical analysis was done using a 1-way ANOVA test. **(B)** Localisation and post-translational modifications of STAT3 in mitochondrial and cytoplasmic fractions of BMDM (n=4). **(C)** Whole cell lysates of BMDM were prepared after 3 h of stimulation with IL-6 or PMT and either used directly as WCL control or subjected to immunoprecipitation with a monoclonal OPA1 antibody. Membranes were immunoblotted using an antibody recognising total STAT3. As input control, a light chain specific antibody against the IP antibody was used. Equal OPA1 levels were verified by using WCL samples and blotting against OPA1 and actin as loading controls (n=4). **(D)** Serine phosphorylation of STAT3 and OPA1 expression was determined in mitochondrial fractions in the presence and absence of the pan-PKC inhibitor gö6983 (n=3). **(E)** Biochemical fractionation and subsequent analysis of mitochondrial and cytoplasmic fractions for deamidated and total levels of Gαq, respectively (n=4). Hsp90 and COXIV were used as purity controls for cytoplasmic and mitochondrial fractions.

To understand how PMT might cause the observed increase in mitochondrial activity, we focussed on PMT-mediated STAT3 activation, because STAT3 is a central player in PMT-mediated signalling (Orth et al., 2007) and is known to have non-genomic functions when it localises in the mitochondria (Garama et al., 2016). Thus, we investigated if PMT treatment would result in serine-phosphorylation of STAT3, the signal that is required for mitochondrial translocation (Wegrzyn et al., 2009). The quantification of the western blots shown in **Figure 3A** documents that PMT, but not M-CSF/RANKL, causes an increase in the phosphorylation of STAT3 on S727. The expression of ETC proteins depends on the ability of the mitochondrial membrane to form extensive cristae and the central regulator for cristae formation is the dynamin kinase OPA1 (Beninca et al., 2014). Both, classical and PMT-induced differentiating osteoclasts showed a substantial increase in OPA1 expression on day 3, but this seemed to be more pronounced after PMT treatment (**Figure 3A**). As *Opa1* has a STAT3 binding site in its promoter (Nan et al., 2017), we investigated the role of PMT-induced STAT3-mediated transcription but did not detect major differences for *Opa1* between M-CSF/RANKL and PMT-treated cells (**Supplementary Figure 4**). This is supported by the observation that both types of differentiating osteoclasts show an increase in OPA1 (**Figure 3A**). Because only PMT is able to trigger STAT3 serine phosphorylation, we investigated if pSer STAT3 translocated into the mitochondria (add space between pSer and STAT3 and remove :). Because RANKL and M-CSF-mediated signalling do not trigger activation of the JAK-STAT pathway, we used the pro-inflammatory and osteoclastogenic STAT3-activating cytokine IL-6 as control. Indeed, we found serine-phosphorylated STAT3 to be localised in the mitochondria after PMT and IL-6 treatment (**Figure 3B**). As there was no difference in the *Opa1* gene induction between RANKL and PMT samples, and only PMT caused mitochondrial STAT3 translocation, we hypothesised that STAT3 might stabilise OPA1 expression through a direct protein-protein interaction. To this end, we performed co-precipitations using an OPA1 antibody and subsequent detection of precipitated STAT3 by immunoblotting. Again, IL-6 was used as a control. **Figure 3C** shows that PMT as well as IL-6 trigger the formation of a direct interaction between OPA1 and STAT3. Protein kinase C (PKC) has been reported to phosphorylate STAT3 and is activated downstream of Gαq (Gartsbein et al., 2006). To see if Gαq-mediated PKC activation might trigger STAT3 serine phosphorylation and translocation, we inhibited PKC with the inhibitor gö6983. This resulted in decreased pSer STAT3 levels, as well as a substantial decrease in OPA1 expression (**Figure 3D**). This suggests that the observed decrease in OPA1 expression after inhibition of PKC is due to the missing interaction between STAT3 and OPA1 in the mitochondria. Using OPA1-deficient MEFs, Beninca et al. found that Gαq supports mitochondrial activity by increasing the stability of the dynamin like GTPase OPA1, a finding which was supported by further studies (Beninca et al., 2014; Del Dotto et al., 2017). Therefore, we examined the localisation and activation of Gαq by PMT and found a preferential mitochondrial localisation and its deamidation at the mitochondria (**Figure 3E**). These data suggest that PMT-mediated Gαq activation and the resulting serine phosphorylation of STAT3 is a key event that eventually allows mitochondrial translocation of pSer STAT3 by mitochondrial carrier proteins (Boengler et al., 2010; Tammineni et al., 2013). In the mitochondria, the previously described stabilisation of OPA1 might therefore be mediated through a direct interaction between OPA1 and STAT3.

### 3.5 PMT enhances mitochondrial respiratory spare capacity

**Figure 4:**
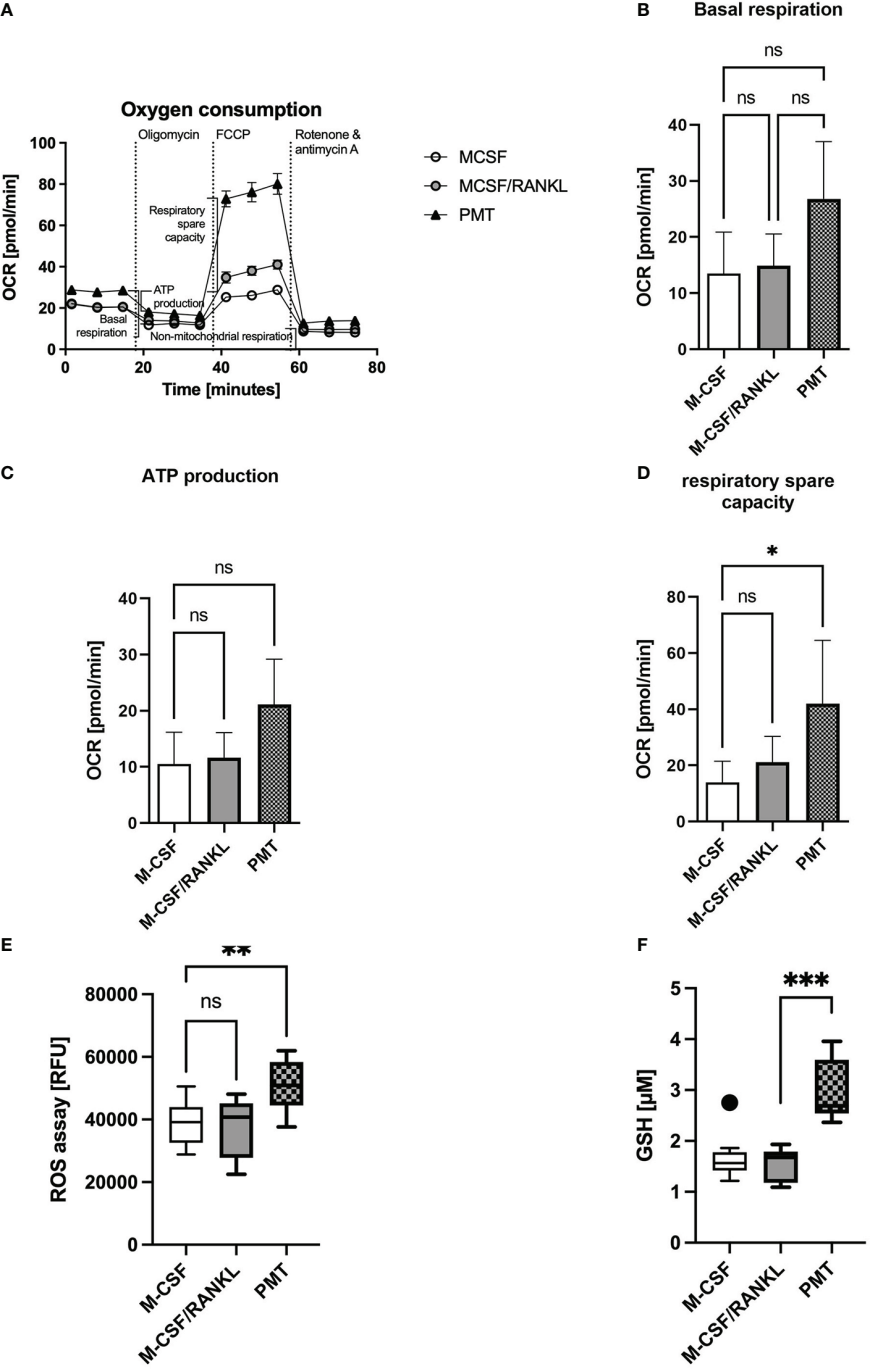
PMT treatment increases mitochondrial respiration. **(A)** Seahorse analysis of mitochondrial activity of BMDM on day 3 of differentiation (Mito Stress Test) (n=3). **(B)** Quantification of basal respiration, **(C)** ATP production and **(D)** respiratory spare capacity. Statistical analysis was done using a Friedman test for paired samples. **(E)** Quantification of ROS production of BMDM on day 3 of osteoclast differentiation (n=5, with triplicates). Statistical analysis was done using a Kruskal-Wallis test on unmatched samples. **(F)** Glutathione levels of BMDM were quantified on day 3 of differentiation and luminescence was measured (n=3, in triplicates). Concentration of GSH was calculated using a standard curve with glutathione (16 μM to 0.125 μM). One outlier was removed by Tukey analysis. Statistical analysis was done using a Kruskal-Wallis test on unmatched samples.

Increased OPA1 protein expression was described to enhance complex I and II activity and the oxygen consumption rate (OCR) and the production of ATP (Nan et al., 2017; Rincon and Pereira, 2018). In addition, mitochondrial STAT3 is known to support cellular respiration through stabilisation of complex I and II (Wegrzyn et al., 2009). Therefore, we investigated if the Gαq-mediated effects on cristae structure and OxPhos expression resulted in a change in OCR. Mitochondrial activity was measured on day 3 of differentiation using a Seahorse mito stress test assay (**Figure 4A**). Here, PMT-treated developing osteoclasts showed an enhanced basal respiration when compared with macrophages and a higher ATP production than cytokine-induced osteoclasts (**Figures 4A-C**), although this effect did not reach statistical significance. Moreover, the respiratory spare capacity was significantly enhanced in PMT-treated differentiating osteoclasts (**Figure 4D**). As both types of differentiating osteoclasts had increased OPA1 levels, enhanced OPA1 expression alone seems to be insufficient to increase the OCR. This observation also suggests that additional mechanisms, e.g. pSer STAT3, are involved in the PMT-mediated increase in OCR. Interestingly, we observed that PMT-treated cells also produce higher amounts of ROS on day3 (**Figure 4E**), which is a secondary messenger for osteoclastogenesis. Due to the constitutive G protein activation, PMT-induced ROS production supports cell differentiation not only at early steps of differentiation as reported for RANKL (Lee et al., 2005). In addition, we observed a significant increase in cellular glutathione levels in PMT-treated cells, which might represent a protective mechanism to avoid cell damage (**Figure 4F**).

### 3.6 *GNAQ* and *OPA1* are overexpressed in arthritis

**Figure 5:**
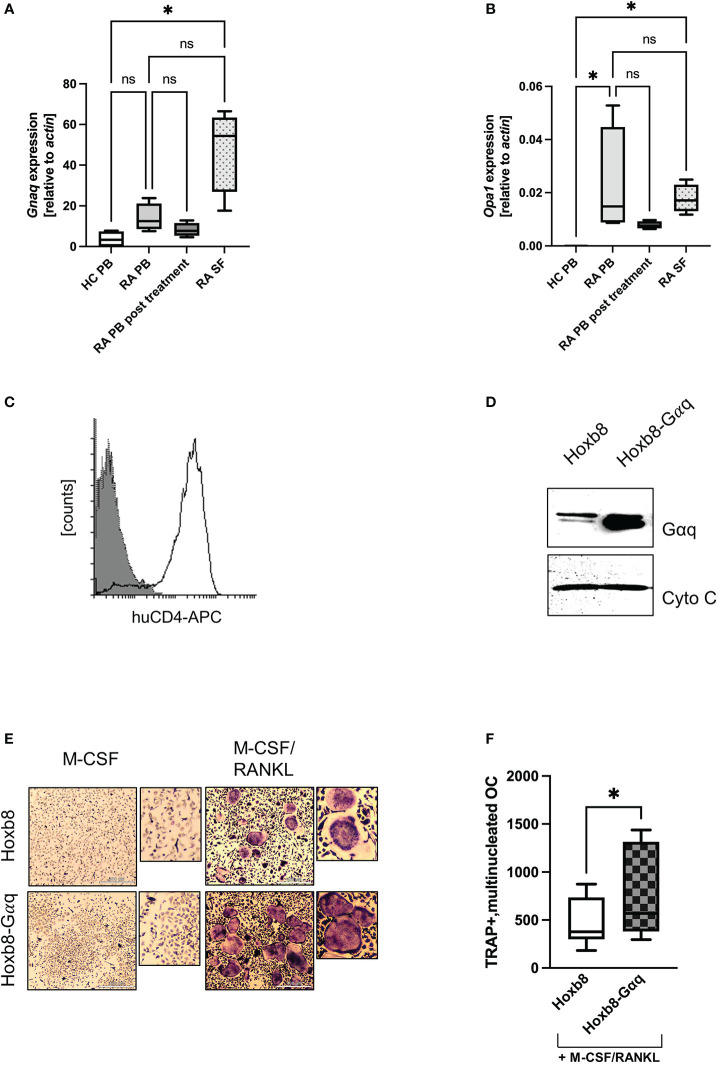
Overexpression of Gαq in ER-Hoxb8 cells causes a hypermetabolic phenotype. **(A)** *GNAQ* and **(B)** *OPA1* expression was quantified by RT-PCR using peripheral blood (PB) from healthy controls and RA patients, as well as synovial fluid from RA patients. In addition, PB of post-treatment RA patients was analysed (n=4). Statistical analysis was done using a Friedman test. **(C)** ER-Hoxb8 cells were retrovirally transduced with murine Gαq and successful transduction was verified by FACS analysis for huCD4. **(D)** Overexpression of Gαq and Cytochrome C as loading control. **(E)** TRAP assays were performed to study the effect of Gαq on OC formation of ER-Hoxb8 cells. Cells were stained for TRAP and multinucleated, TRAP positive cells were quantified in **(F)** (n=4). Statistical significance was evaluated with a 2-way ANOVA test.

The data indicated that Gαq signalling was central to the increased mitochondrial activity. Therefore, we asked whether an increase in Gαq expression might also be observed in patients with increased osteoclastogenic activity, such as RA. For this pathology, it was described that patient-derived macrophages display a phenotype of hyperactivated mitochondrial metabolism (Weyand et al., 2017). When we investigated the expression of *GNAQ* in the peripheral blood from healthy donors and RA patients, we observed a trend towards increased *GNAQ* expression in RA patients compared to the healthy control. This increase in *GNAQ* was significantly higher when synovial fluid samples were used for analysis (**Figure 5A**). Similar results were obtained for *OPA1*, where a significantly increased gene induction in blood and synovial fluid from RA patients was detectable (**Figure 5B**). Again, PB from post-treatment RA patients, who are in disease remission phase, showed decreased levels of *OPA1*. This observation suggests that the heightened expression of *GNAQ* and *OPA1* at the disease site might augment mitochondrial function which plausibly facilitates the enhancement of osteoclastogenesis in the inflamed joints of RA patients. This suggests that Gαq levels influence osteoclastogenesis in RA patients and that the effect of already existing Gαq inhibitors should be investigated in the future.

To prove that the effects observed for treatment with PMT are actually driven by the constitutive activation of Gαq signalling, we retrovirally overexpressed Gαq in ER-Hoxb8 cells (**Figure 5C**), a C57Bl/6-derived cell line that expresses only low amounts of endogenous Gαq (**Supplementary Figure 5A**). **Figure 5D** shows successful transduction by verifying the enhanced Gαq expression levels. As a first read-out, TRAP assays were performed and the number of differentiated cells was determined. **Figures 5E, F** show that Gαq overexpression increased the differentiation into TRAP-positive, multinucleated osteoclasts. The higher variability observed for the number of TRAP-positive, multinucleated ER-Hoxb8-Gαq cells could be caused by the fact that Gαq cells were slower in adhering to the wells, thus having a slower kinetics and a higher number of yet smaller-sized cells.

### 3.7 Gαq overexpression causes a phenotype of hypermetabolic activity

**Figure 6:**
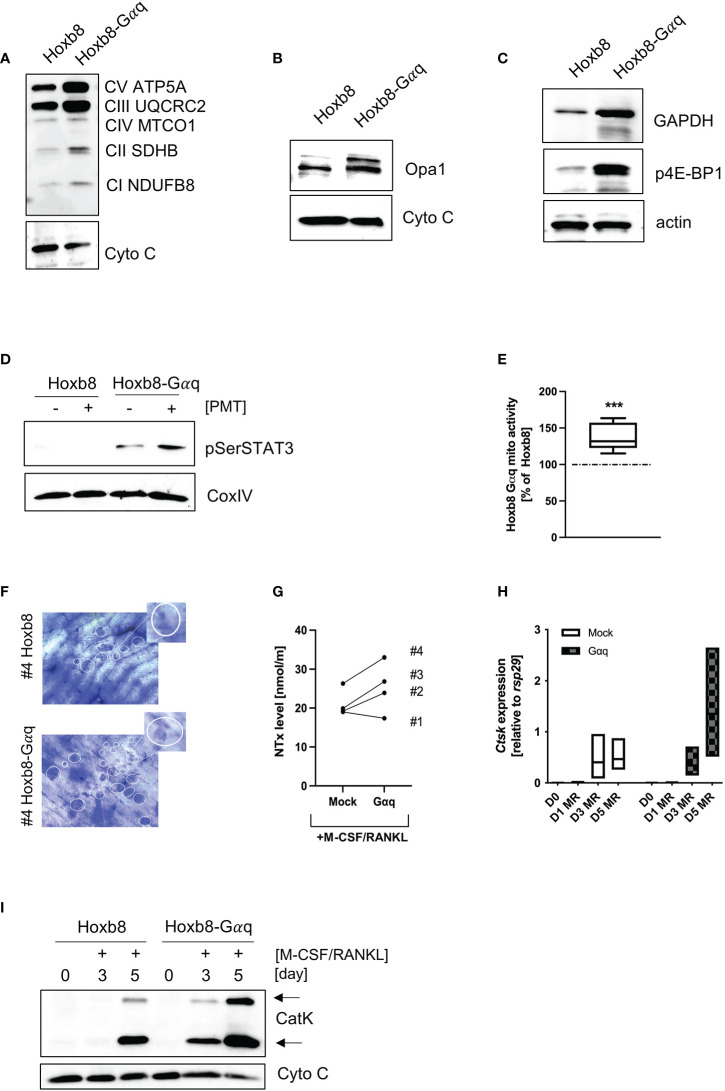
Overexpression of Gαq in ER-Hoxb8 cells mimics the hypermetabolic phenotype. Lysates from mock-transduced and Gαq-transduced ER-Hoxb8 cells were probed for the expression of OxPhos components **(A)**, OPA1 **(B)**, GAPDH and p4E-BP1 **(C)**. Cytochrome C and actin were used as controls (n=3). **(D)** ER-Hoxb8 and ER-Hoxb8-Gαq cells were left untreated or stimulated with 1 nM PMT for 3 hours before lysis. Lysates were probed for pSer STAT3; COXIV was used as a lysate control (n=3). **(E)** Mitochondrial activity was quantified by Mitotracker Deep Red analysis and subsequent evaluation of mfi values (n=5). Statistical analysis was done performing a Wilcoxon test on the raw data before normalisation to ER-Hoxb8 as 100%. **(F)** Bone slices were incubated for 14 days with ER-Hoxb8 and ER-Hoxb8-Gαq cells before staining the bone with toluidine blue. The bone slices shown correspond to #4 of the ELISA shown in **(G)**, where the supernatants from the bone slices were used to quantify the released collagen (day 3) **(H)** RT-PCR analysis of ER-Hoxb8 and ER-Hoxb8-Gαq cells for *Ctsk* induction normalised to *rsp29* on days 0, 1, 3 and 5 (n=3). **(I)** Western Blot analysis of cell extracts from ER-Hoxb8 and ER-Hoxb8-Gαq cells of days 0, 3 and 5 and detection of immature and mature forms of Cathepsin K, using Cytochrome C as a control for equal loading (n=3).

To see whether Gαq overexpression would be sufficient to mimic the phenotype of PMT-induced constitutive Gαq activation, we checked the expression pattern of central signalling molecules. Indeed, we could corroborate an increase in OxPhos expression (**Figure 6A**), OPA1 (**Figure 6B**), GAPDH as well as p4E-BP1 (**Figure 6C**). Also, we observed an increase in pSer STAT3 levels that was caused by overexpression of Gαq and was enhanced by PMT treatment (**Figure 6D**), showing the link between Gαq and the ability to cause serine STAT3 phosphorylation. As expected, we could also observe higher mitochondrial activity (**Figure 6E**). To see if the enhanced metabolic activity would result in increased resorptive activity, we performed RT-PCR analysis for activity-related genes (*Ctsk*, *Mmp9*, *Acp5* and *Dcstamp*), western blot analysis for Cathepsin K expression and cleavage into its active form as well as bone resorption assays and subsequent quantification of released collagen (**Figures 6F–I**; **Supplementary Figures 5B–D**). We observed that Gαq cells were more active than the control cells in bone resorption. Also, Gαq overexpressing cells showed earlier and enhanced expression of Cathepsin K on gene and protein level relative to the control cells indicating that Gαq has the potential to accelerate the process of differentiation into osteoclasts as well as its bone resorptive function. This suggests that not only an accelerated differentiation but also enhanced functional activity plays a role.

## 4 Discussion

Bacterial toxins and pathogenicity factors can have the ability to target and influence mitochondrial processes. Most often this results in a disruption of mitochondria and has therefore been linked to host cell apoptosis. Prominent examples include the listeriolysin O (LLO) of *Listeria monocytogenes* and *Helicobacter pylori* VacA and Toxin AB and B from *Clostridium difficile*, among many others (Jiang et al., 2012; Zhang et al., 2019). However, prevention of programmed cell death or a diversion of antimicrobial strategies have also been reported, which highlights the fact that bacteria might have a benefit from the interaction with mitochondria (Cohen et al., 2018; Mazzoleni et al., 2021). A recent paper by Berger et al. suggests that the interaction between bacteria and host cell mitochondria can have an impact on the immune response through a change in immunometabolic activity (Berger et al., 2017). *Citrobacter rodentium* infection causes gut intestinal epithelial cells to switch their metabolic activity towards aerobic glycolysis, ultimately leading to the establishment of a favourable gut ecosystem and the reduction of other colon-associated anaerobic commensals. Toxigenic *Pasteurella* strains might use PMT to evade an efficient innate immune response by creating a favourable environment where osteoclast differentiation is preferred over macrophage activation (Chakraborty et al., 2017). In this manuscript we investigated the impact of PMT on the metabolic activity of differentiating osteoclasts. As the relationship between macrophages and osteoclasts is important in inflammatory diseases of both increase in activity of PMT-mediated osteoclasts, microbial and auto-immune origin, a better understanding of the molecular details may enhance our perspective on the molecular targets of the disease (Yao et al., 2021).

Osteoclasts have to adapt their metabolic activity throughout their differentiation process to enable cell fusion and resorption of inorganic material (Kubatzky et al., 2018). Not much is known about the pathways that translate variations in metabolic demands into changes of mitochondrial architecture. Loss of mTOR, the master regulator of glycolysis, changes mitochondrial cristae morphology. This is caused by the proteolytic cleavage of OPA1 which eventually results in reduced respiratory capacity (Parra et al., 2014). Conversely, it was found that mitochondrial integrity and activity of the ETC are necessary for effective glycolysis even in cells like neutrophils that do not use the TCA cycle and therefore express only a small number of mitochondria (Amini et al., 2018). This stresses that glycolysis and TCA cycle should not be regarded as separate entities as they are efficiently intertwined. Interestingly, it was shown that the organization of the cytoskeleton also plays a role in the ability of osteoclasts to make use of mitochondrial activity for specific osteoclast functions (Zhang et al., 2018). It was shown that PGC1β-deficiency resulted in the loss of cytoskeletal integrity which resulted in impaired mitochondrial activity and bone resorption. PMT causes a strong activation of cytoskeletal activation through RhoA-mediated stress fibre formation (Orth et al., 2005; Kloos et al., 2015). Whether the observed increase of PMT-mediated osteoclasts in activity is influenced by their specific cytoskeletal organization still needs to be investigated.

Our data show that osteoclasts differentiating with PMT display a distinct phenotype where glycolysis, mTOR pathway and translational activity are strongly activated. As a consequence, expression of OxPhos proteins and mitochondrial activity are elevated. Gαq-dependent phosphorylation of STAT3 is a central step as it promotes mitochondrial translocation and increases mitochondrial activity. Consequently, overexpression of Gαq in ER-Hoxb8 cells was sufficient to mimic this phenotype. Our data suggest that mitoSTAT3 helps restructuring mitochondria through increased expression or stability of OPA1, a GTPase required for efficient cristae folding. Through this, OPA1 increases mitochondrial respiration and helps to maintain functionality of OxPhos (Cogliati et al., 2016). Correspondingly, in Gαq/11-deficient fibroblasts, OPA1 levels as well as the numbers of cristae were reduced, causing narrow junctions and reduced ATP synthesis (Beninca et al., 2014). MitoSTAT3 was described to interact with ETC complexes to support electron flux and increase mitochondrial metabolism (Garama et al., 2016). In cardiomyocytes, mitoSTAT3 enhanced OxPhos activity and acted protectively during ischaemia/reperfusion (Yang and Rincon, 2016) and in cancer this promotes reprogramming from glycolysis to OxPhos (Lee et al., 2019). Our data suggest that this may additionally be supported by OPA1, another well-known mediator of cardiomyocyte resistance to apoptosis. The observed increase in OPA1 expression seems to be mediated through the direct association with mitoSTAT3, as inhibition of STAT3 translocation to the mitochondria decreases, OPA1 expression. This would represent a novel function for mitoSTAT3 that was previously shown to act as a stabilising factor in the mitochondria through its interaction with GRIM-19 of the ETC complex I (Huang et al., 2004; Wegrzyn et al., 2009). Little is known about a possible function of mitoSTAT3 in immune cell regulation. Rincon et al. suggest that IL-21 and IL-6 play a role in mitochondrial functions of STAT3 in lymphocytes. In CD4 T cells, IL-6 causes mitochondrial hyper-polarization and increases mitochondrial calcium. As a consequence, cytosolic calcium levels increase and support persistent cytokine expression (Yang et al., 2015; Rincon and Pereira, 2018). The cytokine RANKL does not activate STAT3 and mitoSTAT3 is therefore not involved in osteoclast formation under physiological conditions. As a consequence, mitochondrial respiration does not reach the high levels observed for PMT-induced osteoclasts (**Figure 4A**). However, in an inflammatory microenvironment, IL-6 is highly expressed by macrophages and potentially triggers STAT3 serine phosphorylation as well as its mitochondrial translocation making it an interesting future target for therapy of inflammatory bone diseases (Li et al., 2022). In RA patient lymphocytes, enhanced nuclear STAT3 activity was observed and inhibition of STAT3 tyrosine phosphorylation or overexpression of the mitoSTAT3 interaction partner GRIM-19 ameliorated RA as it reduced excess glycolytic activity, increased OxPhos activity and reduced the amount of osteoclast stimulating Th17 T cells (Moon et al., 2014; McGarry et al., 2018). Interestingly, PMT treatment not only upregulated ROS through enhanced OxPhos activity, but also lead to a significant increase in glutathione levels and thus limits oxidative stress. A similar phenomenon was observed in cancer cells, where increased mito STAT3 was found to be required for glutathione synthesis (Garama et al., 2015; Lahiri et al., 2021).

Apart from cancer cells, also macrophages from RA patients display a distinct, hypermetabolic phenotype, characterised by the dual activation of glycolysis and OxPhos (Weyand et al., 2017; Zeisbrich et al., 2018). As we had observed that overexpression of Gαq causes a similar hypermetabolic state, we wanted to know if Gαq overexpression might be of pathophysiological relevance in RA. Indeed, we found *Gnaq* to be upregulated in peripheral blood and synovial fluid from RA patients. This is in contrast with other studies that found a lower *GNAQ* expression in RA lymphocytes (Wang et al., 2012). Interestingly, there is also a gender bias as the expression of *GNAQ* increases in an oestrogen-dependent manner in immune cells (Morton et al., 2003). In addition, as outlined above, the metabolic regulation of lymphocytes and macrophages from RA patients is fundamentally different. It is therefore possible that Gαq plays a role in the mitochondrial hyperactivity observed in macrophages but is decreased in lymphocytes for the same reason.

It has been suggested that improving mitochondrial physiology might be an interesting option to reduce inflammation in RA (Castegna et al., 2020), but it can be anticipated that immunometabolic targets need to be addressed in a cell type specific manner. Gαq was reported to be down-regulated in RA patient lymphocytes (Liu et al., 2015). Targeting Gαq in macrophages might therefore be an interesting option that circumvents the dual roles observed for other proteins, such as mTOR, STAT3 or HIF-1α. It is currently discussed to target G protein-coupled receptors (GPCR) signalling by developing inhibitors for their downstream subunits (Campbell and Smrcka, 2018; Kostenis et al., 2020). This has the advantage that receptors that employ more than one heterotrimeric G protein can be targeted pathway specific. GPCRs play critical roles in a number of clinically relevant diseases, such as cancer, cardiovascular diseases as well as infection and inflammation (Jo and Jung, 2016). Chemokine receptors for example determine the spatio-temporal organisation of the leukocyte immune response (Lammermann and Kastenmuller, 2019). Not surprisingly, these receptors have also been implicated in the pathogenesis of autoimmune pathologies like SLE, rheumatoid arthritis and systemic sclerosis, where GPCR activation could be linked to Gαq activation downstream in same cases (Zhang and Shi, 2016). More importantly, GPR91, the receptor for the TCA metabolite succinate, was found to signal *via* Gαq in macrophages (Trauelsen et al., 2021). This GPCR can activate pro- and anti-inflammatory signalling as well as osteoclastic gene expression *via* Gαq-mediated activation of NFATc1 (Guo et al., 2017). GPR91 represents a central molecular switch in RA and correspondingly inhibition or deletion of GPR91 ameliorated the disease (Littlewood-Evans et al., 2016; Saraiva et al., 2018; Velcicky et al., 2020). For Gαq, two bioavailable inhibitors exist at the moment (YM254890, FR900359) (Taniguchi et al., 2003; Schrage et al., 2015). However, only preclinical studies for their effectiveness in melanoma, asthma and thrombosis have been performed to date and the suitability for treatment of autoimmune diseases remains to be investigated. Our data suggest that inhibition of Gαq might be an interesting drug target to modulate cellular metabolic activity.

## Data availability statement

The original contributions presented in the study are included in the article/**Supplementary Material**, further inquiries can be directed to the corresponding author/s.

## Ethics statement

The studies involving human participants were reviewed and approved by Institute Ethics Committee (IEC-490/01.09.2017) of the Department of Transplant Immunology and Immunogenetics, All India Institute of Medical Sciences, New Delhi 1100029, India. The patients/participants provided their written informed consent to participate in this study. The animal study was reviewed and approved by Regierungspräsidium Karlsruhe.

## Author contributions

Conceptualization: KK, KB and FU. Methodology, SC, BH, and KB. Investigation: SC, BH, DY, AH, and JS. Formal Analysis: DS. Resources: TT, SK and TV. Writing – Original Draft, KK and SC. Writing, Review & Editing: KK, SC and BH. Funding Acquisition KK and SC.
