# Supplementary material for: Gαq modulates the energy metabolism of osteoclasts
Source: Front Cell Infect Microbiol. 2023 Jan 9;12:1016299. doi: 10.3389/fcimb.2022.1016299 (PMC9869164; doi:10.3389/fcimb.2022.1016299)
Supplement: Supplementary file 1 [file DataSheet_1.pdf]

1. Supplementary data

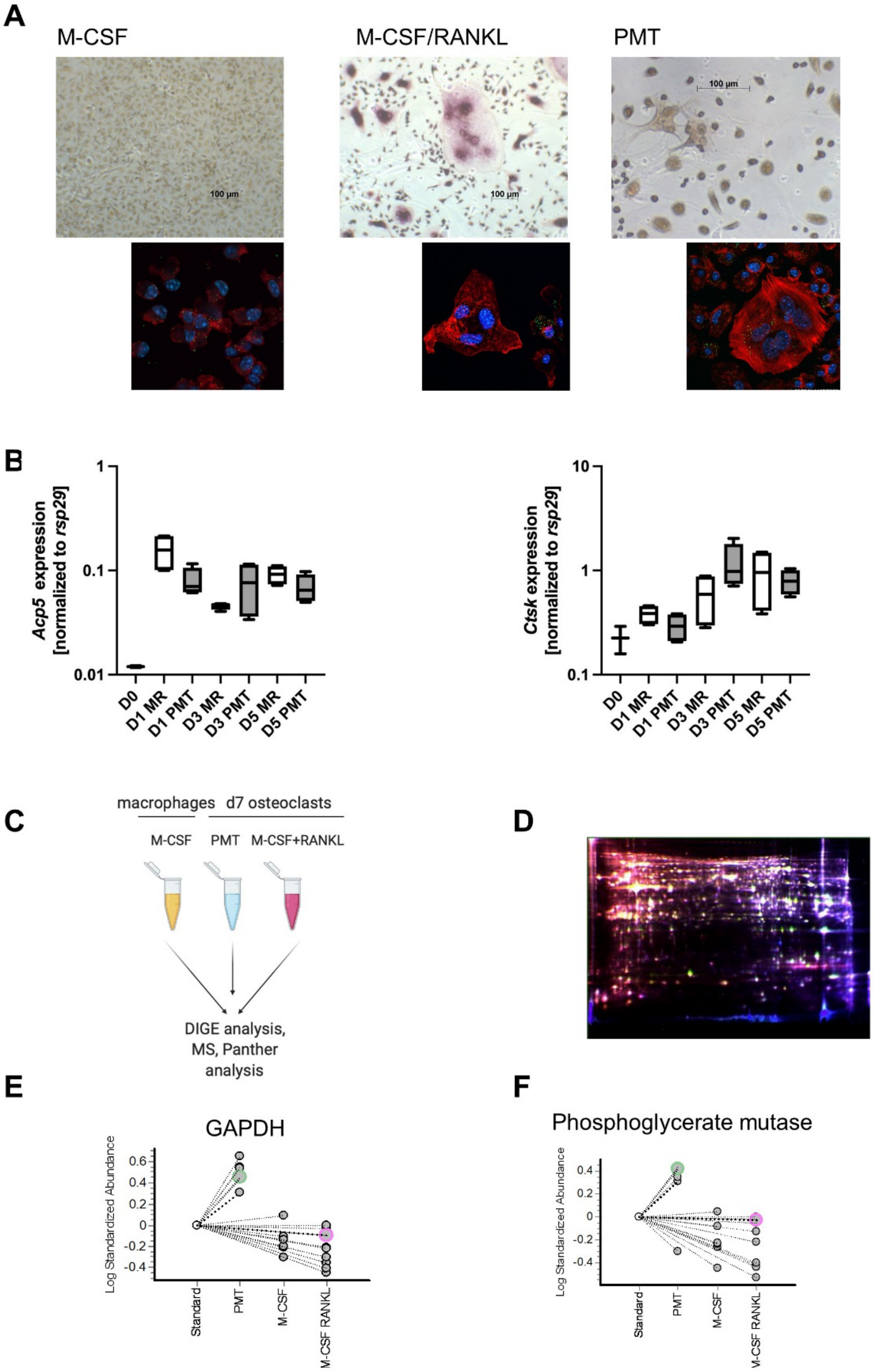

Supp Figure 1:
(A)(B) Analysis of M-CSF/RANKL and PMT-mediated osteoclast differentiation. (A) BMDM were stimulated with M-CSF, M-CSF/RANKL and PMT for 5-7 days as described in the methods section. Cells were fixed and stained for TRAP activity (upper part). For

confocal laser scanning microscopy, cells were enzymatically labelled to show active TRAP (ELF97) (green). Nuclei were stained with DAPI (blue) and the cytoskeleton with TRITC-Phalloidin (red). Representative pictures of stained cells are shown. (B) RT-PCR analysis of the osteoclast marker genes *Ctsk* and *Acp5* during differentiation. (C)(D) DIGE analysis of the proteome of the different stimulation approaches (M-CSF, M-CSF/RANKL and PMT) of BMDM (day7). Shown is a representative DIGE gel with the overlay of Cy2, Cy3, and Cy5 fluorescent dyes. For the horizontal dimension, a pH gradient from 3-10 was used. In the vertical dimension, the proteins were separated according to their molecular weight (10 kDa-150 kDa). M-CSF/RANKL-treated sample were stained with Cy3, PMT-treated samples with Cy5. (E,F)) Examples of significant differentially expressed proteins in M-CSF, M-CSF/RANKL and PMT-treated macrophages. Graphic representation of the fluorescence intensity of two significantly altered protein spots, which were evaluated using the BVA module of the DeCyder software. Protein expression was first normalised against the internal standard and then compared between the different samples and gels. Each circle represents the protein spot in the different gels (n=6; 3 biological and 2 technical replicates).

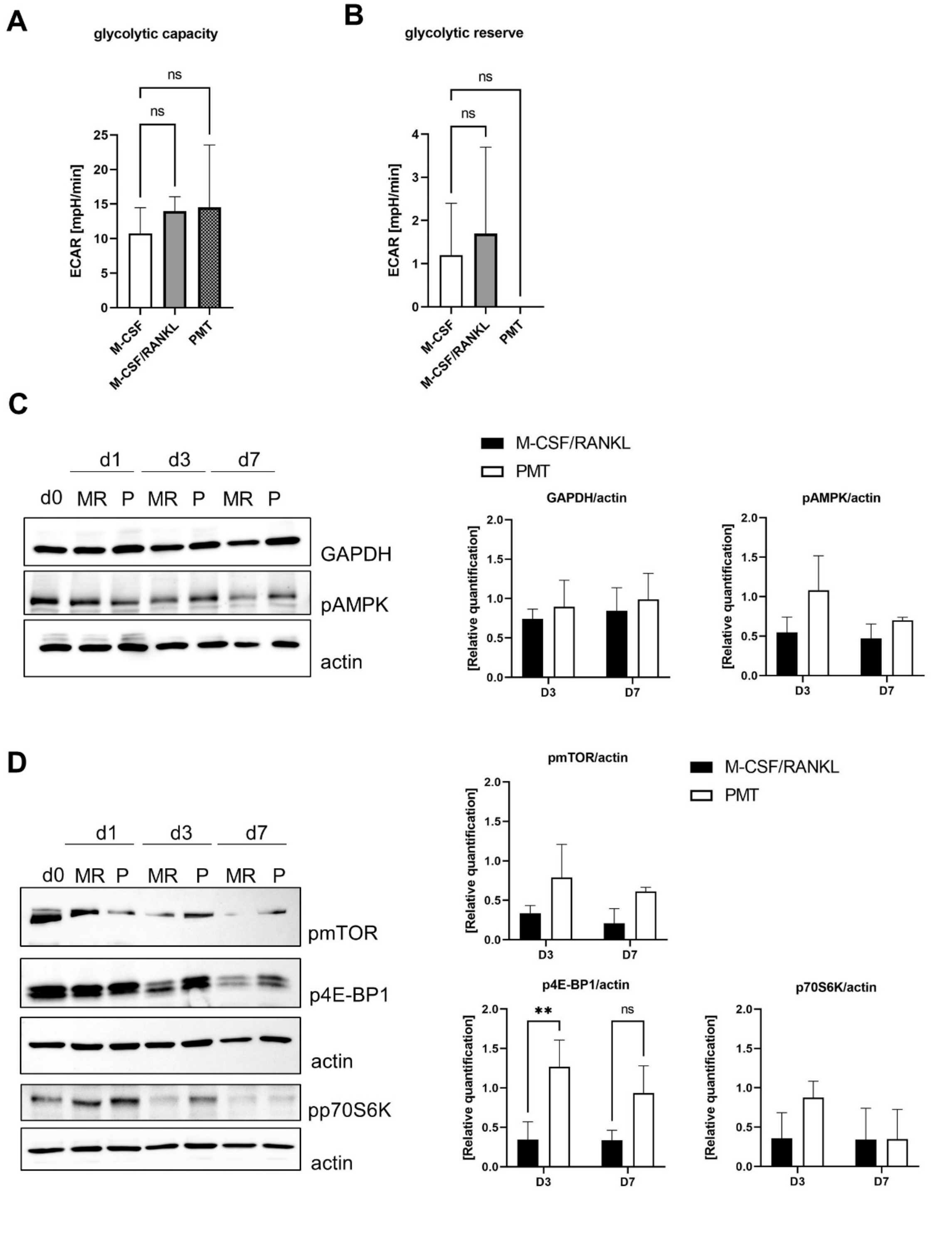

Supp Figure 2:

(A)(B) Glycolysis of BMDM differentiated for 3 days with M-CSF, M-CSF/RANKL or PMT was investigated in a Seahorse glycolysis assay with quantification of the total glycolytic capacity (A) and glycolytic reserve (B) (n=3). For statistical analysis a Friedman test was used. Figure 1A was created using BioRender software. BMDM were stimulated as indicated and lysates from day 0 (untreated), 1, 3 and 7 were probed for the expression of pAMPK and GAPDH (C) or pmTOR, p4E-BP1, pp70S6K, respectively (D). Actin was used as a lysate control (n=3) and quantification of proteins relative to actin is shown. Statistical analysis was performed by 2-way ANOVA.

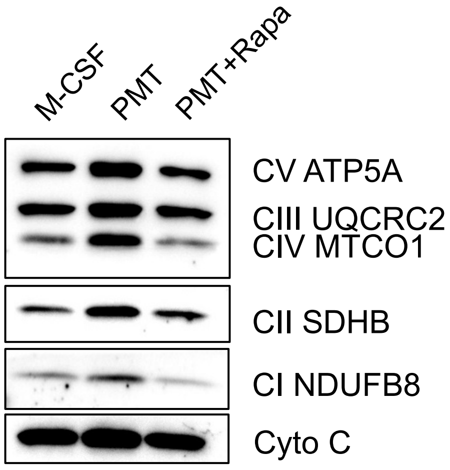

Supp Figure 3:
Expression of OxPhos proteins on day 3 of differentiation of BMDM treated with M-CSF, PMT and PMT in the presence of the mTORC1 inhibitor Rapamycin (10 ng/ml). Cytochrome C was used as control (n=3).

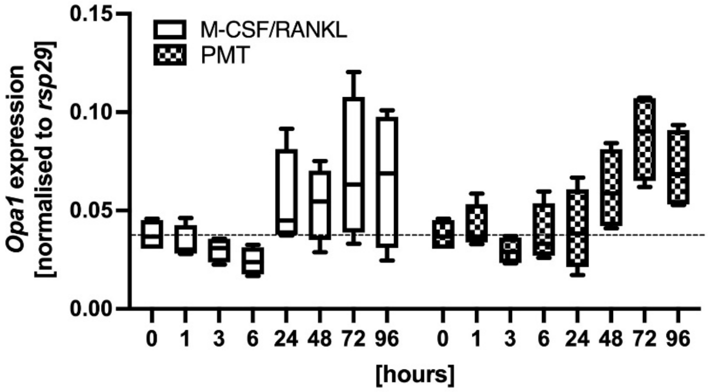

Supp Figure 4:

RT-PCR analysis of *Opa1* gene induction in BMDM treated with M-CSF/RANKL and PMT treated cells for osteoclast differentiation.

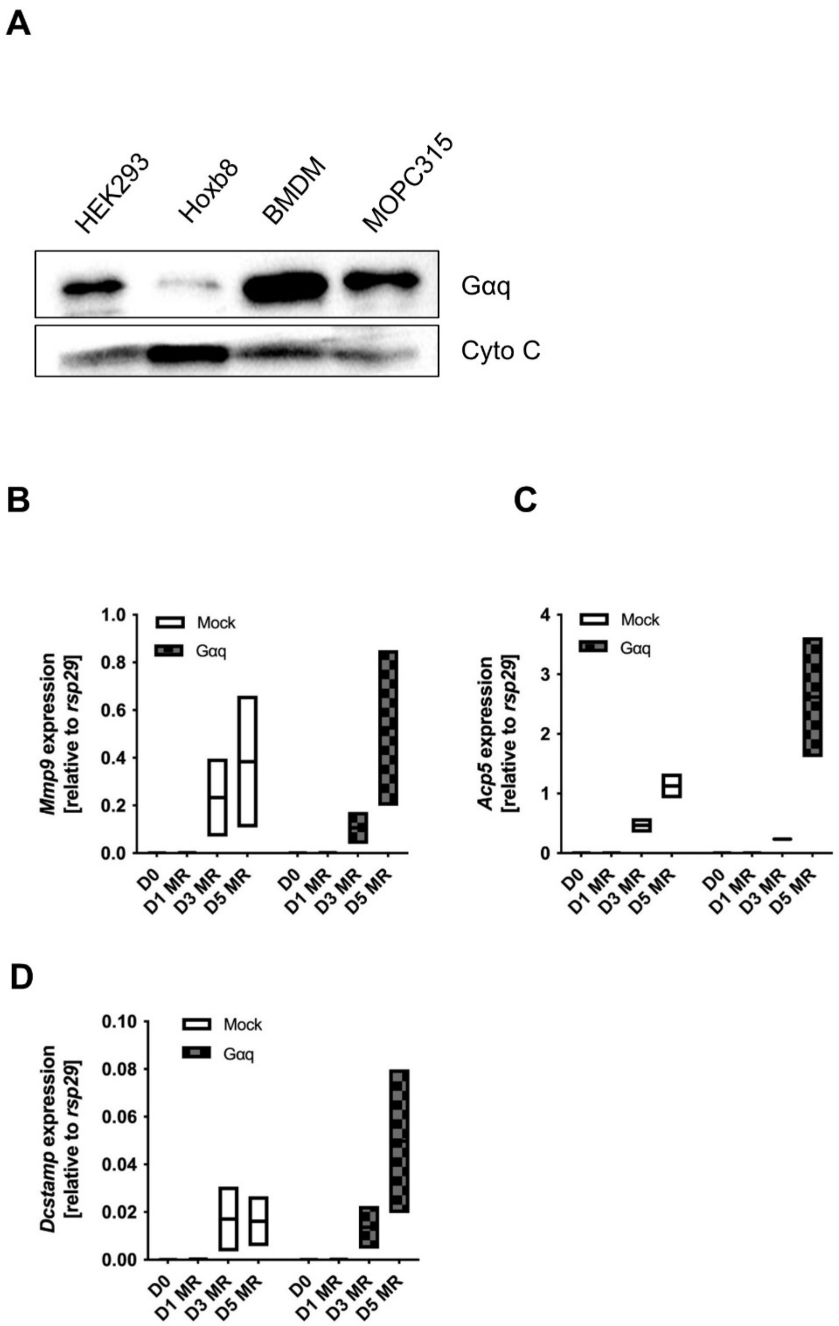

Supp Figure 5:

(A) Western Blot analysis of Gαq expression in BMDM, ER-Hoxb8 and MOPC 315 cells. (B)(C)(D) RT-PCR analysis of *Mmp9*, *Dcstamp and Acp5* gene induction in BMDM treated with M-CSF/RANKL and PMT treated cells during osteoclast differentiation on days 0, 1, 3 and 5 (n=3).

2. Supplementary Table

Table 1: Mass spectrometry data from Orbitrap analysis (original data of 7 most highly regulated spots (n=3 biological replicates with 3 technical replicates each)

Table 1

Orbitrap-Results:

A1

Hit No.	Accession No.	Protein Description
1	gi|70794816	uncharacterized protein LOC433182 [Mus musculus]
2	gi|40254781	rab GDP dissociation inhibitor beta [Rattus norvegicus]
3	gi|158517895	tetratricopeptide repeat protein 38 [Mus musculus]
4	gi|20071682	Lpxn protein [Mus musculus]
5	gi|33859662	synaptic vesicle membrane protein VAT-1 homolog [Mus musculus]
6	gi|27754031	sorting nexin-6 [Mus musculus]
7	gi|19527008	group XV phospholipase A2 precursor [Mus musculus]
8	gi|809561	gamma-actin [Mus musculus]
9	gi|404057	adenylosuccinate synthetase [Mus musculus]
10	gi|30048137	Wdsub1 protein, partial [Mus musculus]
11	gi|4895037	coronin-1 [Mus musculus]
12	gi|1587323	interferon-stimulated gene factor 3gamma
13	gi|14039466	protocadherin-betaO [Mus musculus]
14	gi|14250422	Phosphogluconate dehydrogenase [Mus musculus]
15	gi|6753036	aldehyde dehydrogenase, mitochondrial precursor [Mus musculus]
16	gi|1617402	vasodilator-stimulated phosphoprotein [Mus musculus]
17	gi|26325850	unnamed protein product [Mus musculus]
18	gi|16716569	protease, serine, 1 precursor [Mus musculus]
19	gi|84781771	trypsin 10 precursor [Mus musculus]
20	gi|7513694	IgG Fc binding protein - mouse (fragment)

A2

Hit No.		Protein Description
1	gi|7106255	arginase-1 [Mus musculus]
2	gi|70794816	uncharacterized protein LOC433182 [Mus musculus]
3	gi|6754994	poly(rC)-binding protein 1 [Mus musculus]
4	gi|6754724	26S proteasome non-ATPase regulatory subunit 7 [Mus musculus]
5	gi|50510343	mKIAA0060 protein [Mus musculus]
6	gi|84579893	syntaxin-17 [Mus musculus]
7	gi|14587839	acyl-CoA hydrolase [Mus musculus]
8	gi|10946870	alcohol dehydrogenase [NADP(+)] [Mus musculus]

9	gi|13435924	Aldolase C, fructose-bisphosphate [Mus musculus]
10	gi|30425302	ras association domain-containing protein 2 [Mus musculus]
11	gi|6671539	fructose-bisphosphate aldolase A isoform 2 [Mus musculus]
12	gi|12859782	unnamed protein product [Mus musculus]
13	gi|12843914	unnamed protein product [Mus musculus]
14	gi|1167510	TI-225 [Mus musculus]
15	gi|387397	epidermal keratin subunit I, partial [Mus musculus]
16	gi|398168	keratin 2 epidermis [Mus musculus]
17	gi|1184246	vesicle membrane protein [Mus musculus]
18	gi|22164776	keratin, type II cytoskeletal 79 [Mus musculus]
19	gi|16716569	protease, serine, 1 precursor [Mus musculus]
20	gi|3642647	delta-aminolevulinic acid dehydratase, partial [Mus musculus]
21	gi|5442444	actin-related protein complex 1b [Mus musculus]

A3

Hit No.		Protein Description
1	gi|6996913	annexin A2 [Mus musculus]
2	gi|198845	lipocortin I [Mus musculus]
3	gi|6679937	glyceraldehyde-3-phosphate dehydrogenase [Mus musculus]
4	gi|6755568	schlafen 1 [Mus musculus]
5	gi|70794816	uncharacterized protein LOC433182 [Mus musculus]
6	gi|7106255	arginase-1 [Mus musculus]
7	gi|59624979	three prime repair exonuclease 1 [Mus musculus]
8	gi|786001	aldose reductase [Mus musculus]
9	gi|21450083	regulation of nuclear pre-mRNA domain-containing protein 1A [Mus musculus]
10	gi|10946870	alcohol dehydrogenase [NADP(+)] [Mus musculus]
11	gi|6753404	CCAAT/enhancer-binding protein beta [Mus musculus]
12	gi|74191644	unnamed protein product [Mus musculus]
13	gi|6754524	L-lactate dehydrogenase A chain isoform 1 [Mus musculus]
14	gi|21312520	dihydropteridine reductase [Mus musculus]
15	gi|26324736	unnamed protein product [Mus musculus]
16	gi|12859782	unnamed protein product [Mus musculus]
17	gi|1167510	TI-225 [Mus musculus]
18	gi|6753060	annexin A5 [Mus musculus]
19	gi|17066601	peroxisomal acyl-CoA thioesterase 2 [Mus musculus]
20	gi|6671539	fructose-bisphosphate aldolase A isoform 2 [Mus musculus]
21	gi|14250422	Phosphogluconate dehydrogenase [Mus musculus]
22	gi|16716569	protease, serine, 1 precursor [Mus musculus]
23	gi|191765	alpha-fetoprotein, partial [Mus musculus]
24	gi|12843046	unnamed protein product [Mus musculus]
25	gi|19527388	ubiquitin thioesterase OTUB1 [Mus musculus]
26	gi|6688788	putative transmembrane glycoprotein [Mus musculus]

A4

Hit No.		Protein Description
1	gi|6679937	glyceraldehyde-3-phosphate dehydrogenase [Mus musculus]
2	gi|6996913	annexin A2 [Mus musculus]
3	gi|70794816	uncharacterized protein LOC433182 [Mus musculus]
4	gi|7106255	arginase-1 [Mus musculus]
5	gi|6754524	L-lactate dehydrogenase A chain isoform 1 [Mus musculus]
6	gi|12835827	unnamed protein product [Mus musculus]
7	gi|12843046	unnamed protein product [Mus musculus]
8	gi|3329498	heterogenous nuclear ribonucleoprotein A2/B1 [Mus musculus]

A5

Hit No.		Protein Description
1	gi|6755965	voltage-dependent anion-selective channel protein 2 [Mus musculus]
2	gi|19073015	citrate lyase beta subunit [Mus musculus]
3	gi|70794816	uncharacterized protein LOC433182 [Mus musculus]
4	gi|7949037	delta(3,5)-Delta(2,4)-dienoyl-CoA isomerase, mitochondrial precursor [Mus musculus]
5	gi|6679937	glyceraldehyde-3-phosphate dehydrogenase [Mus musculus]
6	gi|50510343	mKIAA0060 protein [Mus musculus]
7	gi|63100260	Mercaptopyruvate sulfurtransferase [Mus musculus]
8	gi|359279904	tropomyosin alpha-3 chain isoform 3 [Mus musculus]
9	gi|27413160	carbonyl reductase [NADPH] 3 [Mus musculus]
10	gi|26353732	unnamed protein product [Mus musculus]
11	gi|6755963	voltage-dependent anion-selective channel protein 1 [Mus musculus]
12	gi|112363072	actin-related protein 2/3 complex subunit 2 [Mus musculus]
13	gi|1304157	78 kDa glucose-regulated protein [Mus musculus]
14	gi|6753284	caspase-3 [Mus musculus]
15	gi|1292952	3-hydroxy-3-methylglutaryl-CoA lyase [Mus musculus]
16	gi|12846304	unnamed protein product [Mus musculus]
17	gi|862463	uridine phosphorylase [Mus musculus domesticus]
18	gi|33416530	Annexin A4 [Mus musculus]
19	gi|12843046	unnamed protein product [Mus musculus]
20	gi|16716569	protease, serine, 1 precursor [Mus musculus]
21	gi|191765	alpha-fetoprotein, partial [Mus musculus]
22	gi|2505940	26S proteasome, non-ATPase subunit [Mus musculus]
23	gi|309319	heat shock protein 70 cognate [Mus musculus]
24	gi|7106255	arginase-1 [Mus musculus]
25	gi|12832065	unnamed protein product [Mus musculus]

A6

Hit No.		Protein Description
1	gi|12844989	unnamed protein product [Mus musculus] (phosphoglycerate mutase 1)
2	gi|7242142	COP9 signalosome complex subunit 7a isoform 1 [Mus musculus]

3	gi|6753556	cathepsin D precursor [Mus musculus]
4	gi|70794816	uncharacterized protein LOC433182 [Mus musculus]
5	gi|1103844	steroid dehydrogenase [Mus musculus]
6	gi|6755965	voltage-dependent anion-selective channel protein 2 [Mus musculus]
7	gi|19526463	endoplasmic reticulum resident protein 29 precursor [Mus musculus]
8	gi|16716569	protease, serine, 1 precursor [Mus musculus]
9	gi|6679937	glyceraldehyde-3-phosphate dehydrogenase [Mus musculus]
10	gi|1526541	14-3-3 eta [Mus musculus]
11	gi|4877287	deoxyguanosine kinase 1 [Mus musculus]
12	gi|84781771	trypsin 10 precursor [Mus musculus]
13	gi|191765	alpha-fetoprotein, partial [Mus musculus]
14	gi|227293	cathepsin B

A7

Hit No.		Protein Description
1	gi|165377185	apoptosis-associated speck-like protein containing a CARD [Mus musculus]
2	gi|309202	mouse preprocathepsin B [Mus musculus]
3	gi|13278412	EG433182 protein [Mus musculus]
4	gi|2897818	huntingtin interacting protein-2 [Mus musculus]
5	gi|13435747	Rho GDP dissociation inhibitor (GDI) alpha [Mus musculus]
6	gi|2746723	cathepsin S precursor [Mus musculus]
7	gi|1407663	SH3P2, partial [Mus musculus]
8	gi|49868	put. beta-actin (aa 27-375) [Mus musculus]
9	gi|14198355	Proteasome (prosome, macropain) subunit, beta type 4 [Mus musculus]
10	gi|10179944	phosphoglycerate mutase 1 [Mus musculus]
11	gi|16716569	protease, serine, 1 precursor [Mus musculus]
12	gi|21313476	GTP-binding protein SAR1b [Mus musculus]
13	gi|12848935	unnamed protein product [Mus musculus]
14	gi|6679937	glyceraldehyde-3-phosphate dehydrogenase [Mus musculus]
15	gi|12843046	unnamed protein product [Mus musculus]
16	gi|7546551	Chain A, Cocrystal Structure Of The Messenger Rna 5' Cap-Binding Protein (Eif4e) Bound To 7-Methyl-Gdp
17	gi|26330162	unnamed protein product [Mus musculus]
18	gi|7657031	5'(3')-deoxyribonucleotidase, cytosolic type [Mus musculus]
19	gi|15489456	Ift80 protein, partial [Mus musculus]
