# Supplementary material for: Gαq modulates the energy metabolism of osteoclasts
Source: Front Cell Infect Microbiol. 2023 Jan 9;12:1016299. doi: 10.3389/fcimb.2022.1016299 (PMC9869164; doi:10.3389/fcimb.2022.1016299)
Supplement: Supplementary file 2 [file Table_1.pdf]

Key Resources Table

REAGENT or RESOURCE	SOURCE	IDENTIFIER
**Antibodies**		
Rabbit monoclonal anti-pAMPKα (Thr172)	Cell Signaling	2531
Mouse monoclonal anti-GAPDH	Proteintech	60004-1-Ig
Rabbit monoclonal anti-β-Actin	Proteintech	20536-1-AP
Rabbit monoclonal anti-p4E-BP1 (Thr37/46)	Cell Signaling	2855
Rabbit monoclonal anti-pmTOR (Ser2448)	Cell Signaling	5536
Rabbit polyclonal anti-pp70S6K	Cell Signaling	9205
Mouse monoclonal anti-total OXPHOS cocktail	Abcam	Ab110413
Rabbit polyclonal monoclonal anti-Hsp90	Cell Signaling	4874
Mouse monoclonal anti-Gαq	Santa Cruz	sc-136181
Rabbit monoclonal anti-CoxIV	Cell Signaling	4850
Rat anti-Gαq(QE)	Kamitani et al., 2011	N/A
Rabbit monoclonal anti-OPA1	Cell Signaling	80471
Rabbit monoclonal anti-pStat3 (Ser727)	Cell Signaling	94994
Rabbit monoclonal anti-STAT3	Cell Signaling	4904
Rabbit polyclonal anti-Cytochrome C	Proteintech	10993-1-AP
Goat polyclonal anti-mouse IgG light chains	Jackson/ Dianova	115-035-174
Goat monoclonal anti-mouse IgG, HRP-linked	Cell Signaling	7076
Goat monoclonal anti-Rabbit IgG, HRP-linked	Cell Signaling	7074
Mouse anti-human CD4 APC	Miltenyi Biotec	130-113-210
**Biological Samples**		
Peripheral blood and Synovial fluid of Rheumatoid arthritis; Peripheral blood of Healthy subjects	All India Institute Of Medical Science, New Delhi	Institute Ethics Committee (IEC-490/01.09.2017)
**Chemicals, peptides, and recombinant proteins**		
MitoTracker® Deep Red FM	Cell Signaling	8778
Gö 6983	Merck	365251
Pasteurella multocida toxin (PMT)	Chakraborty et al., 2017	N/A
YM 254890	Biomol	AG-CN2-0509
Human IL-6	Miltenyi Biotec	N/A
Recombinant Mouse M-CSF Protein	Biotechne	416-ML
Recombinant Mouse TRANCE/RANK L/TNFSF11 Protein	Biotechne	462-TR
Elf 97 phosphatase substrate	Thermo Fisher	E6588
DAPI	Thermo Fisher	MP01306
TRITC-conjugated Phalloidin	Sigma Aldrich	P1951
CellROX™ Deep Red Reagent	Thermo Fisher	C10422
Puromycin	Sigma-Aldrich	P8833
Protein A/G plus agarose	Santa Cruz	sc-2003
β-estradiol	Sigma-Aldrich	E2758
β-mercaptoethanol	PAN Biotech	P07-05020
**Critical commercial assays**		
Click-iT™ HPG Alexa Fluor™ 488 Protein Synthesis Assay Kit	Thermo Scientific	C10428
QPRO-BCA Kit Standard	Cyanagen Srl	PRTD1,0500
Total ROS Assay Kit 520 nm	Thermo Scientific™	88-5930-74

GeneJET RNA Purification Kit (human data)	Thermo Scientific™	K0732
Revert Aid First strand cDNA synthesis kit (human data)	Thermo Scientific™	K1621
PowerUp™ SYBR™ Green Master Mix (human data)	Applied Biosystems™	A25741
innuPREP RNA Mini Kit 2.0	Analytikjena	845-KS-2040250
cDNA synthesis kit	Biozym Scientific	331470L
2x qPCRBIO SyGreen Mix Hi-ROX	PCR Biosystems	PB20.12-01
DNAeasy Blood and Tissue Kit	Qiagen	69504
Mitochondria Isolation Kit for Mammalian cells	Thermo Fisher	89874
ProGel Tris/Glycin gel 4-20% 1.0 mm	Anamed	TG42012
WESTAR ηC Ultra 2.0	Cyanagen Srl	XLS075,0100
Leukocyte acid phosphatase (TRAP) Kit	Sigma-Aldrich	387A-1KT
Bovine Cross linked N-telopeptide of type I collagen, NTX ELISA Kit	MYBioSource	MBS1602302
Toluidine Blue O	Sigma-Aldrich	T3260
ProFection® Mammalian Transfection System	Promega	E1200
GSH/GSSG-Glo™ Assay	Promega	V6611
Amersham CyDye DIGE Fluor Minimal Labeling Kit	Cytiva	25801065
Sodium pyruvate	Thermo Fisher	N/A
Penicillin-Streptomycin Solution 100X	Anprotec	AC-AB-0024
Seahorse XFp Cell Mito Stress Test Kit	Agilent	N/A
Seahorse XFp Cell Glycolysis Stress Test Kit	Agilent	N/A
Seahorse XF Base Medium	Agilent	N/A
Lymphoprep	Axis-Shield	1114544
D(+)Glucose	Sigma-Aldrich	N/A
HEPES	Agilent	N/A
L-glutamine	Thermo Fisher	N/A
FCS	Biochrome	N/A
RPMI 1640 w/ stable Glutamine	Anprotec	AC-LM-0056
DMEM High Glucose w/ stable Glutamine w/ Sodium Pyruvate	Anprotec	AC-LM-0013
**Experimental models: Cell lines**		
Mouse cell line: L-929	ATCC	CCL-1
Mouse cell line: ER-Hoxb8	Wang et al., 2006	N/A
Mouse cell line: LGM3 (muGM-CSF)	Zal et al., 1994	N/A
Mouse cell line: MOPC 315	ATCC	TIB-23
Human cell line: Phoenix-Eco	ATCC	SD-3444
**Experimental models: Organisms/strains**		
Mouse: C57BL/6	Janvier-labs	N/A
**Recombinant DNA**		
pMX-IRES-CD4-Puro	Gündogdu et al., 2010	N/A
pMX-Gq-IRES-CD4-Puro	This paper	N/A
pCl-eco	Naviaux et al., 1996	N/A
**Software and algorithms**		
FACSDiva™	BD Biosciences	https://www.bdbiosciences.com/
bioRENDER	bioRENDER	https://biorender.com/
DeCyder 2D Version 7.0	Amersham Bioscience	N/A
Flowing Software 2	TURKU BIOSCIENCE	https://bioscience.fi/

ChemoStar TS	Intas Sciennce Imaging	ChemoStar PC ECL & Fluorescence Imager - Chemiluminescence - Fluorescence / WesternBlotting - Intas
Fiji	Image J	https://imagej.net/software/fiji/
LAS AF software	Leica	Leica Mikrosysteme Vertrieb GmbH, Wetzlar
Rebel Microscope	ECHO	https://discover-echo.com/
PANTHER Classification System	PANTHER	http://www.pantherdb.org/index.jsp
Seahorse XF Cell Mito Stress Test Report Generator 3.0.11	Agilent	N/A
Seahorse XF Glycolysis Stress Test Report Generator 4.0	Agilent	N/A
GraphPad Prism 9	GraphPad	https://www.graphpad.com/

**Primers**	**Sequence 5'→3'**
Rsp29, Mouse Forward	AGCCGACTCGTTCCTTTCTC
Rsp29, Mouse Reverse	CGTATTTGCGGATCAGACC
Slc2a1, Mouse Forward	CAGTTCGGCTATAACACTGGTG
Slc2a1, Mouse Reverse	GCCCCCGACAGAGAAGATG
Pgc1b, Mouse Forward	CTCCAGGCAGGTTCAACCC
Pgc1b, Mouse Reverse	GGGCCAGAAGTTCCCTTAGG
Opa1, Mouse Forward	ACCTTGCCAGTTTAGCTCCC
Opa1, Mouse Reverse	TTGGGACCTGCAGTGAAGAA
Mmp9, Mouse Forward	CAGCCGACTTTTGTGGTCTTC
Mmp9, Mouse Reverse	CGGTACAAGTATGCCTCTGCCA
Ctsk, Mouse Forward	AGGGAAGCAAGCACTGGATA
Ctsk, Mouse Reverse	GCTGGCTGGAATCACATCTT
Acp5, Mouse Forward primer	TTCCAGGAGACCTTTGAGGA
Acp5, Mouse Reverse primer	GGTAGTAAGGGCTGGGGAAG
Dcstamp, Mouse Forward primer	AAAACCCTTGGGCTGTTCTT
Dcstamp, Mouse Reverse primer	GTTCCTTGCTTCTCTCCACG
mtDNA, Mouse Forward primer	CTAGAAACCCCGAAACCAAA
mtDNA, Mouse Reverse primer	CCAGCTATCACCAAGCTCGT
nucDNA B2M, Mouse Forward primer	ATGGGAAGCCGAACATACTG
nucDNA B2M, Mouse Reverse primer	CAGTCTCAGTGGGGGTGAAT
GNAQ Human Forward primer	CTCTGGAGTCCATCATGGCG
GNAQ Human Reverse primer	CCACTCTCTCCTGTCCCGAG
OPA1 Human Forward Primer	GCCACTTCCTGGGTCATTCC
OPA1 Human Reverse Primer	CAGACCTCACAGGCCACAG
ACTB Human Forward Primer	ATATGAGATGCGTTGTTA
ACTB Human Reverse Primer	AAGTATTAAGGCGAAGAT

Kamitani, Shigeki, et al. "Enzymatic actions of Pasteurella multocida toxin detected by monoclonal antibodies recognizing the deamidated α subunit of the heterotrimeric GTPase Gq." The FEBS journal 278.15 (2011): 2702-2712.

Chakraborty, S., Kloos, B., Harre, U., Schett, G., & Kubatzky, K. F. (2017). Pasteurella multocida toxin triggers RANKL-independent osteoclastogenesis. Frontiers in immunology, 8, 185.
Wang, G. G., Calvo, K. R., Pasillas, M. P., Sykes, D. B., Häcker, H., & Kamps, M. P. (2006). Quantitative production of macrophages or neutrophils ex vivo using conditional Hoxb8. Nature methods, 3(4), 287-293.
Naviaux, Robert K., Eugenia Costanzi, Martin Haas, and Inder M. Verma. "The pCL vector system: rapid production of helper-free, high-titer, recombinant retroviruses." Journal of virology 70, no. 8 (1996): 5701-5705.
Gündogdu, M. S., Liu, H., Metzdorf, D., Hildebrand, D., Aigner, M., Aktories, K., ... & Kubatzky, K. F. (2010). The haematopoietic GTPase RhoH modulates IL3 signalling through regulation of STAT activity and IL3 receptor expression. Molecular cancer, 9(1), 1-13.
Zal T, Volkmann A, Stockinger B., Mechanisms of tolerance induction in major histocompatibility complex class II-restricted T cells specific for a blood-borne self-antigen. J Exp Med. 1994 Dec 1;180(6):2089-99.
Malik, A. N., Czajka, A., & Cunningham, P. (2016). Accurate quantification of mouse mitochondrial DNA without co-amplification of nuclear mitochondrial insertion sequences. Mitochondrion, 29, 59-64.
